# Phosphorylation-Dependent Interactions between Crb2 and Chk1 Are Essential for DNA Damage Checkpoint

**DOI:** 10.1371/journal.pgen.1002817

**Published:** 2012-07-05

**Authors:** Meng Qu, Bing Yang, Li Tao, John R. Yates, Paul Russell, Meng-Qiu Dong, Li-Lin Du

**Affiliations:** 1 College of Biological Sciences, China Agricultural University, Beijing, China; 2 National Institute of Biological Sciences, Beijing, China; 3 Department of Cell Biology, The Scripps Research Institute, La Jolla, California, United States of America; 4 Department of Molecular Biology, The Scripps Research Institute, La Jolla, California, United States of America; The University of North Carolina at Chapel Hill, United States of America

## Abstract

In response to DNA damage, the eukaryotic genome surveillance system activates a checkpoint kinase cascade. In the fission yeast *Schizosaccharomyces pombe*, checkpoint protein Crb2 is essential for DNA damage-induced activation of downstream effector kinase Chk1. The mechanism by which Crb2 mediates Chk1 activation is unknown. Here, we show that Crb2 recruits Chk1 to double-strand breaks (DSBs) through a direct physical interaction. A pair of conserved SQ/TQ motifs in Crb2, which are consensus phosphorylation sites of upstream kinase Rad3, is required for Chk1 recruitment and activation. Mutating both of these motifs renders Crb2 defective in activating Chk1. Tethering Crb2 and Chk1 together can rescue the SQ/TQ mutations, suggesting that the main function of these phosphorylation sites is promoting interactions between Crb2 and Chk1. A 19-amino-acid peptide containing these SQ/TQ motifs is sufficient for Chk1 binding *in vitro* when one of the motifs is phosphorylated. Remarkably, the same peptide, when tethered to DSBs by fusing with either recombination protein Rad22/Rad52 or multi-functional scaffolding protein Rad4/Cut5, can rescue the checkpoint defect of *crb2Δ*. The Rad22 fusion can even bypass the need for Rad9-Rad1-Hus1 (9-1-1) complex in checkpoint activation. These results suggest that the main role of Crb2 and 9-1-1 in DNA damage checkpoint signaling is recruiting Chk1 to sites of DNA lesions.

## Author Summary

To preserve the integrity of genomic DNA, eukaryotic cells use a genome surveillance system to detect DNA damage and send a signal to prevent cell division before DNA repair has been completed. This signal transduction mechanism is called checkpoint signaling and is conserved from yeasts to humans. A key checkpoint component is the protein kinase Chk1, which is activated in response to DNA damage. In fission yeast, the activation of Chk1 requires a number of upstream checkpoint proteins including Crb2, but their exact roles and mechanisms of action are unclear. Here we show that Crb2 interacts with Chk1 when Crb2 is phosphorylated at two conserved N-terminal positions. This phosphorylation-dependent interaction is critical for Chk1 relocalization to damaged DNA and Chk1 activation. By tethering a Crb2 peptide sufficient for Chk1 binding to DNA lesions, we demonstrated that the main role of Crb2 and 9-1-1 complex in checkpoint signaling is to bring Chk1 to sites of DNA damage, where it can be activated.

## Introduction

Maintaining genome integrity requires the DNA damage checkpoint signaling pathways, which typically involve a protein kinase cascade. In vertebrates, residing at the top of the signaling pathways are two members of the phosphatidylinositol 3-kinase-like protein kinase (PIKK) family, ATM (ataxia-telangiectasia, mutated) and ATR (ATM and Rad3 related protein kinase) [1], [2]. The activation of ATM and ATR upon DNA damage leads to the phosphorylation and activation of downstream effector kinases Chk2 and Chk1, which then regulate a myriad of cellular processes including cell cycle progression [3].

In the fission yeast *Schizosaccharomyces pombe*, the ATM ortholog Tel1 plays a minor role in checkpoint signaling at DSBs except when DNA end resection is inhibited [4]. The major checkpoint signaling activity is provided by the ATR ortholog Rad3, which is essential for the signaling through both Chk1 and Chk2 (called Cds1 in fission yeast) [5]–[7]. The activation of Cds1 requires the checkpoint mediator protein Mrc1 [8], [9]. The role of Mrc1 in checkpoint signaling is to recruit Cds1 to stalled replication forks, and this recruitment function relies on multiple Rad3-depedent phosphorylation sites on Mrc1, which serve as docking sites for Cds1 [10]. Acting in between Rad3 and Chk1 is the checkpoint mediator protein Crb2 [11]. Loss of Crb2 completely abolishes Chk1 activation but does not affect Cds1 activation. The requirement for a checkpoint mediator acting upstream of Chk1 is universally conserved. In the budding yeast *Saccharomyces cerevisiae*, the ortholog of Crb2, scRad9, is also vital for the activation of Chk1 [12], [13]. Intriguingly, in vertebrates, the ortholog of Crb2 and scRad9, 53BP1, is dispensable for Chk1 activation. Instead, vertebrate Chk1 activation involves a different mediator, Claspin, which shares no significant homology with Crb2 or scRad9 [14].

The spatial regulation of Crb2 in response to DSBs has served as an instructive model for understanding the influence of chromatin modification on checkpoint mediator localization. When DSBs are generated by ionizing radiation (IR), Crb2 rapidly forms microscopically detectable IR-induced foci (IRIF), and these foci overlap with DSB markers [15]. IRIF formation by Crb2 requires two types of histone modifications: one is histone H2A C-terminal tail phosphorylation catalyzed redundantly by Rad3 and Tel1 kinases [16]; the other is H4-K20 methylation catalyzed by Set9 methyltransferase [17]. Both types of modifications promote histone-Crb2 interactions. Phosphorylated H2A binds to the tandem BRCT domains in the C-terminus of Crb2, which also mediate Crb2 dimerization [18], [19]. K20-methylated H4 interacts with the tandem Tudor domains located N-terminally to the BRCTs [20]. Disrupting either histone-Crb2 interaction dramatically diminishes Crb2 IRIF but only partially impairs checkpoint signaling, indicating the existence of an alternative pathway of recruiting Crb2 to DSBs that does not involve large-scale interactions with chromatin. Indeed, by examining the relocalization of Crb2 to a persistent DSB induced by the HO endonuclease, we previously identified a histone modification-independent Crb2 recruitment pathway, which requires an interaction between Crb2 and Rad4/Cut5 [21].

Rad4/Cut5 is a multi-BRCT domain protein with dual functions in DNA replication and DNA damage checkpoint signaling [22]. The N-terminal tandem BRCT domains in Rad4/Cut5 mediate the interaction with Crb2 [11], and this interaction requires the Crb2-T215 residue, a cyclin-dependent kinase (CDK) phosphorylation site [21], [23]. The second pair of BRCT domains in Rad4/Cut5 mediates an interaction with Rad9 (unrelated to scRad9), which is a subunit of the 9-1-1 checkpoint clamp complex [24]. As the 9-1-1 complex can directly associate with DNA at sites of DSBs [25], the interactions between Rad9 and Rad4/Cut5, and between Rad4/Cut5 and Crb2, provide a means to recruit Crb2 independently of histone-Crb2 interactions.

Compared to our knowledge on how Crb2 is targeted to sites of DNA damage, much less is known about the molecular mechanism by which Crb2 mediates Chk1 activation. Crb2 is hyperphosphorylated upon DNA damage in a Rad3-dependent manner [11], but the Rad3-dependent phosphorylation sites on Crb2 have not been identified. It has also been shown by yeast two-hybrid assay and co-immunoprecipitation under overexpression conditions that Crb2 can interact with Chk1 [11], [26], but the functional significance of such interactions remains unknown. In *S. cerevisiae*, the role of scRad9 in Chk1 activation is also not well understood, except that an N-terminal region of scRad9 is required [13]. In vertebrates, phosphorylated Claspin interacts with the kinase domain of Chk1 to facilitate its activation by ATR [27]–[31]. Due to the lack of homology between Claspin and Crb2/scRad9, it is uncertain whether a common mechanism exists for mediators acting upstream of Chk1.

In this study, we show that Crb2 directly interacts with Chk1 in a phosphorylation dependent manner. Two neighboring SQ/TQ motifs in Crb2, which are consensus sites for ATM/ATR kinases, are critical for Crb2-Chk1 interactions, Chk1 relocalization to DSBs, and DNA damage-induced checkpoint activation. Tethering a Chk1-binding Crb2 peptide to sites of DSBs can bypass endogenous Crb2 and 9-1-1 complex for checkpoint activation, suggesting that the main function of these proteins in DNA damage checkpoint activation is recruiting Chk1.

## Results

### Crb2-dependent accumulation of Chk1 kinase at DSBs

When DSBs occur, the checkpoint mediator Crb2 accumulates at sites of DSBs, forming nuclear foci detectable by fluorescence microscopy [15], [21]. However, it is unclear to what extent its downstream effector Chk1 is concentrated at DSBs.

**Figure 1:**
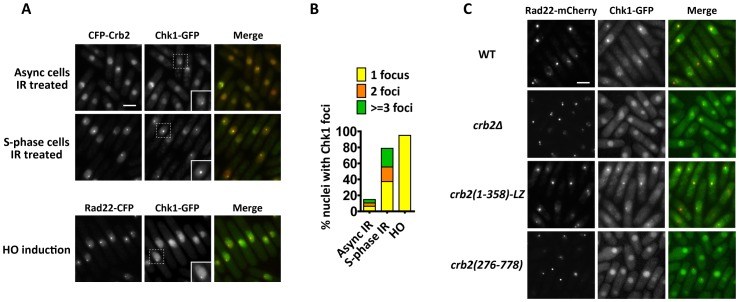
DNA damage-induced Chk1 focus formation requires the N-terminal 275 amino acids of Crb2. (A) Chk1-GFP forms nuclear foci at IR- and HO-induced DSBs. For IR treatment, cells expressing Chk1-GFP and CFP-Crb2 were either treated with 80 Gy IR and incubated for 3 h, or first arrested in early-S phase by a 4-h treatment of 20 mM hydroxyurea (HU), and then treated with 80 Gy IR before releasing into HU-free medium and incubated for 3 h (S-phase IR treatment). For HO endonuclease induction, cells expressing Chk1-GFP and Rad22-CFP were shifted to thiamine-free medium for 16 h to induce the expression of HO, which is under the control of the thiamine-repressible *nmt1* promoter. Strains used were DY6498 and DY6502. Bar, 5 µm. Inset, higher magnification of cells containing Chk1 foci. (B) Quantitation of Chk1 foci in (A). About 200 nuclei were scored for each condition. (C) The N-terminal region of Crb2 is required for Chk1-GFP foci. Cells expressing Chk1-GFP and Rad22-mCherry in wild type (WT), *crb2Δ*, *crb2(1–358)-LZ* or *crb2(276–778)* background were challenged with S-phase IR treatment as in (A). Strains used were DY6498, DY6497, DY6499 and DY6500. Bar, 5 µm.

To test if Chk1 relocalizes to DSBs, we examined the subcellular distribution of C-terminally GFP-tagged Chk1 (Chk1-GFP) by live cell imaging. Expressed from the endogenous promoter, Chk1-GFP is fully functional, as demonstrated by its ability to confer wild-type levels of DNA damage resistance (Figure S1A). Chk1-GFP displayed diffuse nuclear signal in the absence of DNA damage (Figure S1B). IR induced the formation of Chk1-GFP nuclear foci in asynchronized cells (15% of nuclei contained foci), and these foci significantly overlapped with CFP-Crb2 foci in the same cells (Figure 1A). Synchronizing cells to early S-phase with the DNA synthesis inhibitor hydroxyurea (HU) followed by IR exposure and release from HU block (S-phase IR treatment) led to higher frequency (79% of nuclei contained foci) and stronger intensity of Chk1 foci compared to IR treatment of asynchronized cells (Figure 1A and 1B). S-phase IR treatment is expected to result in irreparable DSBs, due to the lack of homologous recombination templates if DSBs occur in unreplicated genomic regions. Such persistent DSBs are known to cause high-level recruitment of Crb2 in a Rad4/Cut5-dependent, histone modification-independent manner [21]. Besides IR, we also used the HO endonuclease to generate a specific DSB in the genome [15]. In response to HO expression, Chk1-GFP formed a single distinct nuclear focus in the majority of the cells, and HO-induced Chk1 foci were completely colocalized with foci formed by a DSB repair protein, Rad22 (homolog of budding yeast Rad52) (Figure 1A and 1B). Similar to the S-phase IR treatment, HO endonuclease cleavage also induces irreparable DSBs, as both sister chromatids are cut by the enzyme. Due to prolonged G2 arrest, both S-phase IR treatment and HO expression led to significant cell elongation, which serves as a useful readout for checkpoint activation (Figure 1A). Together, our observations suggest that Chk1 is recruited to DSBs, and the level of recruitment is enhanced at persistent DSBs.

Chk1 activation requires the upstream kinase Rad3-Rad26, the 9-1-1 checkpoint clamp, the clamp loader Rad17, Rad4/Cut5, and the mediator protein Crb2 [5], [11]. To examine the genetic requirement of DSB-induced Chk1 focus formation, the genes encoding upstream checkpoint factors were individually deleted in strains expressing Chk1-GFP. IR-induced Chk1 foci were not observed in *rad3Δ*, *rad9Δ* and *crb2Δ* cells (Figure S2 and Figure 1C), suggesting that Chk1 relocalization is regulated by the same upstream factors controlling its activation. We only examined asynchronized cells for *rad3Δ* and *rad9Δ* mutants, because S-phase synchronization by HU cannot be performed due to the lack of replication checkpoint.

### Crb2(1–358)-LZ is sufficient for Chk1 focus formation

To understand how Crb2 facilitates Chk1 relocalization, we examined Chk1 focus formation in *crb2Δ* cells expressing truncated forms of Crb2, Crb2(1–358)-LZ or Crb2(276–778). Crb2(1–358)-LZ, which lacks the histone-binding Tudor domains and BRCT domains, but is supplemented with a heterologous leucine zipper (LZ) dimerization motif, forms nuclear foci at persistent DSBs by binding to Rad4/Cut5 [21]. Crb2(276–778), on the other hand, forms transient IRIF in a histone modification-dependent manner [21]. *crb2(1–358)-LZ* cells challenged with S-phase IR treatment formed Chk1 foci with dynamics similar to wild type (Figure 1C). In contrast, no Chk1 foci were observed in *crb2(276–778)* cells (Figure 1C). As previously reported [21], Crb2(1–358)-LZ was sufficient for checkpoint activation that led to G2 arrest and cell elongation, whereas Crb2(276–778) failed to mediate a checkpoint response and cells entered mitosis with unrepaired DSBs (Figure 1C). Thus, the structure-function relationship for the role of Crb2 in Chk1 relocalization parallels that for the checkpoint function of Crb2, namely, the first 275 amino acids of Crb2 is necessary and Crb2(1–358)-LZ is sufficient.

### Two conserved SQ/TQ motifs in the N-terminal region are crucial for Crb2 function

**Figure 2:**
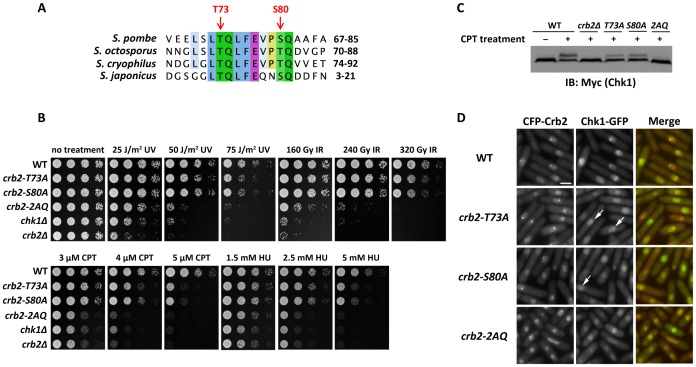
Two conserved SQ/TQ motifs in the N-terminal region of Crb2 are essential for Chk1 recruitment and activation. (A) Sequence alignment of *S. pombe* Crb2 and its orthologs from three other fission yeast species revealed two conserved neighboring SQ/TQ motifs in the N-terminal region of Crb2. The positions of the two motifs in *S. pombe* Crb2 are labeled on top. (B) Mutations in Crb2 SQ/TQ cluster resulted in DNA damage hypersensitivity. Fivefold serial dilutions of cells were spotted on YES plates and incubated at 30°C. Photos were taken 2 d later for untreated, UV-treated, IR-treated and CPT-containing plates. The HU-containing plates were photographed 3 d later. Strains used were LD195, LD346, DY377, DY369, DY370 and DY371. (C) DNA damage-induced Chk1 phosphorylation is defective in Crb2 SQ/TQ cluster mutants. Cells were untreated or treated with 20 µM CPT for 2 h. Cell lysates were separated on SDS-PAGE and probed with an anti-Myc antibody by immunoblotting. Strains used were DY377, LD195, DY369, DY370 and DY371. (D) Mutations in Crb2 SQ/TQ cluster diminished Chk1 foci but not Crb2 foci. Cells expressing Chk1-GFP and CFP-Crb2 were challenged with S-phase IR treatment as in Figure 1A and examined by fluorescence microscopy. Arrows indicate dim Chk1 foci in *crb2-T73A* and *crb2-S80A* cells. Strains used were DY6503, DY6504, DY6505 and DY6506. Bar, 5 µm.

To identify the sequence elements important for Chk1 recruitment and activation in Crb2(1–358)-LZ, sequences of *S. pombe* Crb2 and its homologs from three other fission yeast species were inspected. The N-terminal region of Crb2 lacks significant homology even among these closely related homologs, except for some short stretches of conserved amino acids. One of these short conserved stretches contains an invariant LTQLFE motif followed by an SQ or TQ motif two amino acids downstream (Figure 2A). Previous studies have suggested that clustered SQ/TQ motifs, often phosphorylated by ATM/ATR kinases upon DNA damage, can bridge protein-protein interactions and thereby play important roles in DNA damage response [32]. Thus, we hypothesized that these two conserved SQ/TQ motifs may be involved in the checkpoint function of Crb2.

To test the functional importance of these two SQ/TQ motifs, we substituted one or both of the phosphorylatable residues in these motifs, threonine 73 (T73) and serine 80 (S80), with alanine(s). The two single-residue mutants, denoted as *crb2-T73A* and *crb2-S80A*, displayed mild sensitivity to various types of DNA damage (Figure 2B). They were significantly more sensitive than wild type when treated with higher doses of UV, HU, and CPT, but were much more resistant than either *chk1Δ* or *crb2Δ* at all doses tested. The strain with both T73 and S80 mutated, denoted as *crb2-2AQ*, on the other hand, showed much stronger sensitivity than the single-residue mutants. It appeared to be as sensitive to HU and CPT as *chk1Δ*, and only slightly more resistant to UV and IR than *chk1Δ* (Figure 2B and Figure S3A). The strong synergistic effect of combining the two mutations suggests that these two SQ/TQ motifs may play partially redundant roles in the checkpoint function of Crb2. In a *cdc25-22* block-and-release assay, irradiated *crb2-2AQ* cells entered mitosis as soon as *crb2Δ* cells upon releasing from a G2 block, suggesting a strong defect in checkpoint arrest (Figure S4A). In contrast, both *crb2-T73A* and *crb2-S80A* delayed the mitotic entry significantly, although not as long as the wild type (Figure S4A). To analyze Chk1 phosphorylation and activation, we then examined the DNA damage-induced mobility shift of Chk1 on SDS-PAGE [5]. Chk1 extracted from DNA-damage-treated wild-type cells showed two bands, the upper one corresponding to the phosphorylated form of Chk1 and the lower one corresponding to the unmodified form (Figure 2C and Figure S3B). Only the lower band was observed in either *crb2Δ* or *crb2-2AQ* (Figure 2C and Figure S3B). Consistent with the milder sensitivity and checkpoint defect of single-residue mutants, Chk1 phosphorylation in *crb2-T73A* or *crb2-S80A* was still detectable but weaker than wild type (Figure 2C and Figure S3B). Together, these results suggest that this conserved stretch of residues with two SQ/TQ motifs, which we will thereafter refer to as the SQ/TQ cluster, plays a critical role in Chk1 activation.

### *crb2-2AQ* mutations abrogate DSB–induced focus formation by Chk1 but not Crb2

To understand how the SQ/TQ cluster contributes to Chk1 activation, we examined whether the mutations at the SQ/TQ cluster affect the DNA damage-induced relocalization of Chk1-GFP. To simultaneously monitor the localization of Crb2 in the same cells, we used strains expressing CFP-tagged Crb2 as the only version of Crb2. Upon IR treatment of S-phase cells, Chk1-GFP formed distinct nuclear foci in cells expressing wild-type CFP-Crb2, and these foci completely overlapped with Crb2 foci (Figure 2D). The frequencies of detecting Chk1 foci dramatically decreased in cells expressing Crb2-T73A or Crb2-S80A, and only in a small minority of these cells (about 3%) could we see very faint Chk1 foci, which were also colocalized with Crb2 foci. No Chk1 foci could be detected in cells expressing Crb2-2AQ. In contrast to the strong reduction of Chk1 focus formation, the three mutant forms of Crb2 themselves showed robust focus formation like wild-type Crb2 (Figure 2D). To rule out the possibility that an effect on Crb2 recruitment was masked by the redundancy between the two Crb2 recruitment pathways, we examined the localization of Crb2(1–358)-LZ and found that its focus formation was also unaffected by the 2AQ mutations (Figure S5). Thus, the Crb2 SQ/TQ cluster is not important for the relocalization of Crb2 itself, but rather specifically controls the accumulation of Chk1 at DSBs.

In agreement with the checkpoint defect detected by the *cdc25-22* block-and-release assay and the inability to support Chk1 phosphorylation, *crb2-2AQ* cells did not elongate after the S-phase IR treatment and displayed the “cut” (cell untimely torn) phenotype (Figure 2D, Figure S4B and S4C), indicating a severe defect in G2 arrest. In contrast, cells expressing Crb2-T73A or Crb2-S80A became substantially elongated, consistent with their partial proficiency in Chk1 phosphorylation. We speculate that Chk1 molecules were recruited to DSBs in *crb2-T73A* or *crb2-S80A* cells at a level high enough for partial checkpoint activation but too low to be clearly distinguished from the diffuse nucleoplasmic Chk1-GFP signal.

### Crb2 SQ/TQ cluster is phosphorylated *in vivo*

**Figure 3:**
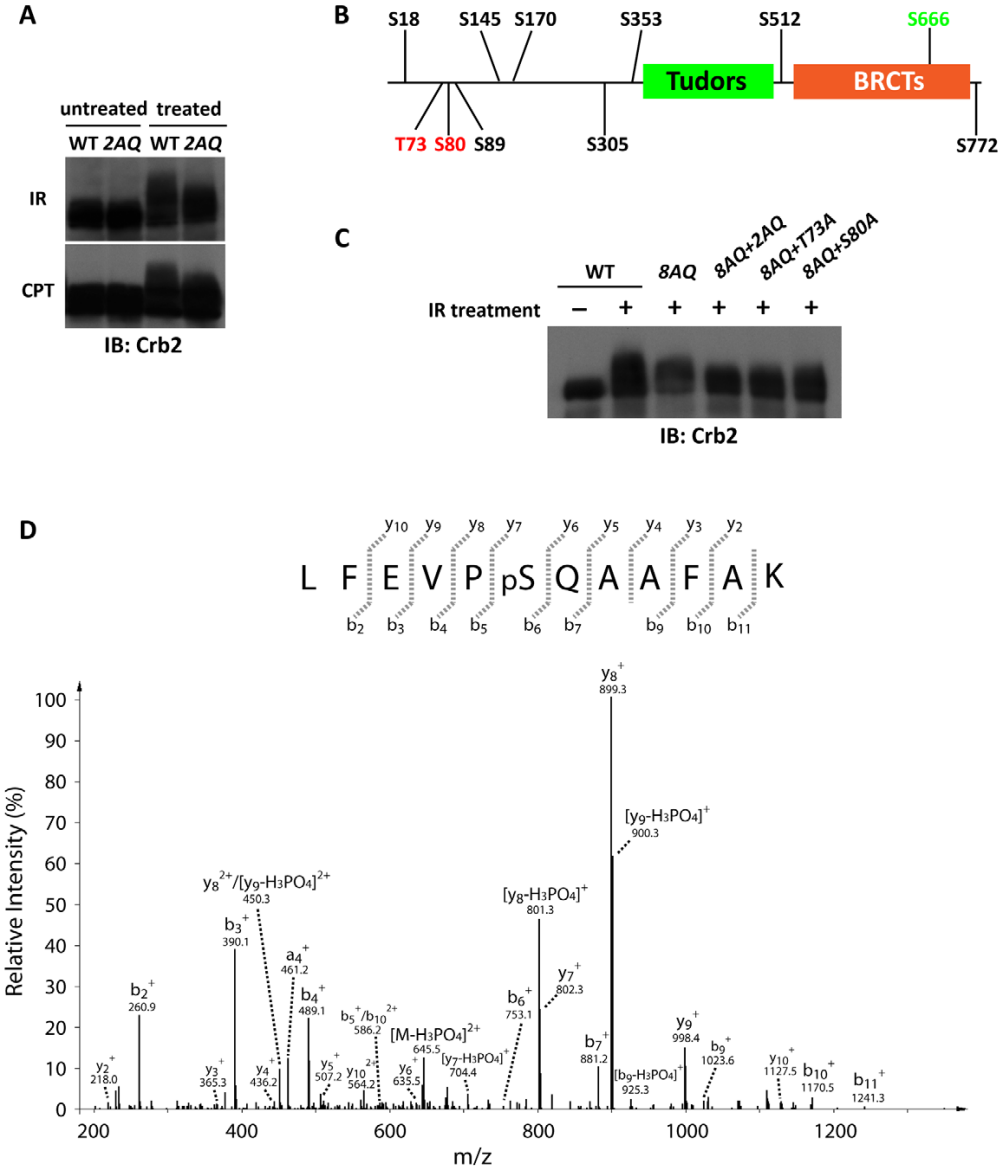
Crb2 SQ/TQ cluster is phosphorylated *in vivo*. (A) *crb2-2AQ* mutations diminished DNA damage-induced Crb2 gel mobility shift. Cells were untreated, or treated with 400 Gy IR or 20 µM CPT. Cell lysates were separated on SDS-PAGE and probed with anti-Crb2 antibody by immunoblotting. Strains used were DY376 and DY383. (B) Locations of eleven SQ/TQ motifs in Crb2. The two conserved residues in the SQ/TQ cluster (T73 and S80) are shown in red, the S666 residue essential for BRCT-mediated dimerization is shown in green, and the other eight SQ/TQs are in black. (C) Mutating the eight SQ/TQs depicted in black in (B) reduced IR-induced Crb2 mobility shift, which could be further attenuated by SQ/TQ cluster mutations. Cells were untreated, or treated with 400 Gy IR. Cell lysates were separated on SDS-PAGE and probed with anti-Crb2 antibody by immunoblotting. Strains used were DY6839, DY6840, DY6843, DY6841 and DY6842. (D) Mass spectrometry analysis showed that S80 residue in Crb2 is phosphorylated. TAP-tagged Crb2 was affinity-purified from cells treated with IR, digested and subjected to mass spectrometry analysis. Phosphorylation of Crb2 at S80 was identified by three overlapping peptides. A representative collision induced dissociation (CID) spectrum of the doubly charged peptide LFEVP[pS]QAAFAK is shown. The strain used was LD412.

Crb2 is known to undergo DNA damage-induced hyperphosphorylation, which manifests as mobility shift on SDS-PAGE [11], [18], [26]. To assess whether the SQ/TQ cluster contributes to Crb2 phosphorylation, we examined the DNA damage-induced mobility shift of Crb2. The 2AQ mutations significantly reduced but did not abolish the mobility shift of Crb2 induced by IR or camptothecin (CPT) (Figure 3A). We hypothesized that other SQ/TQ motifs may contribute to the residual shift in 2AQ mutant, as there are a total of 11 SQ/TQ motifs in Crb2 (Figure 3B). Thus, we mutated all remaining SQ/TQ motifs except S666, which plays a critical structural role at the Crb2 dimer interface and is unlikely to be a phosphorylation site [19], [26]. The resulting 8AQ mutant did not show any DNA damage sensitivity (Figure S6), suggesting that T73 and S80 are the only functionally important ATM/ATR consensus sites. Compared to wild-type Crb2, the 8AQ mutant displayed a less pronounced IR-induced shift, which could be further reduced by the mutations at T73 and/or S80 (Figure 3C). Residual mobility shift was still observed with the 10AQ (8AQ+2AQ) mutant, indicating that DNA damage-induced phosphorylation may also occur on non-SQ/TQ sites. The contribution of T73 and S80 to Crb2 mobility shift suggests that they are phosphorylated in vivo after DNA damage. Because Crb2 mobility shift is dependent on Rad3 [11], Rad3 is most likely the kinase phosphorylating these sites.

To obtain more direct evidence on the phosphorylation of the SQ/TQ cluster, we attempted to create phosphorylation-specific antibodies as well as tried to use an anti-phospho-SQ/TQ antibody. However, we failed to detect Crb2 phosphorylation with these reagents, presumably because the antibodies did not have sufficiently high titers. We then resorted to mass spectrometry for detecting phosphorylation sites on Crb2. TAP-tagged Crb2 purified from cells exposed to IR was digested by three different proteases to increase the peptide coverage. Using a 12-step multidimensional protein identification technology (MudPIT) procedure [33], we identified the phosphorylation of S80 residue (Figure 3D). The failure to detect T73 phosphorylation by mass spectrometry could be due to the difficulty of generating suitable peptides covering that site.

### *In vitro* interactions between Chk1 and Crb2 peptides phosphorylated at T73 and/or S80

**Figure 4:**
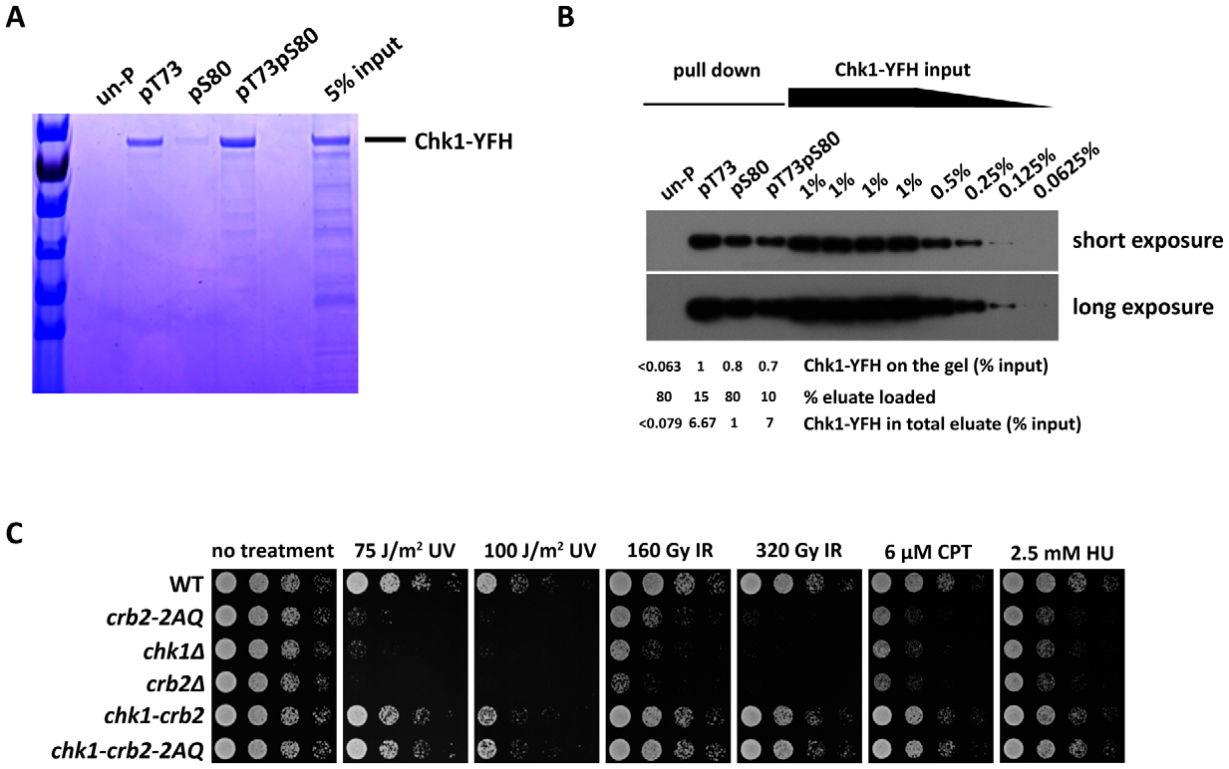
The essential function of the Crb2 SQ/TQ cluster is to mediate a phosphorylation-dependent interaction with Chk1. (A and B) A 19-amino-acid peptide containing the two conserved SQ/TQ motifs, Crb2(67–85), can bind directly to Chk1 when either T73 or S80 is phosphorylated. Chk1 tagged at its C-terminus with YFP-Flag-His6 (YFH) tag was immunoprecipitated from fission yeast cell extract with anti-Flag beads, eluted with Flag peptide and incubated separately with four types of biotin-labeled Crb2(67–85) peptide, un-phosphorylated (un-P), phosphorylated on T73 alone, S80 alone or both. Peptides were pulled down by streptavidin Dynabeads and eluted by boiling in SDS loading buffer. (A) In one experiment, eluates and input of the peptide pull-down assay were analyzed by 4%–20% SDS-PAGE followed by Coomassie staining. (B) In another experiment, eluates and serial dilutions of Chk1-YFH input were examined by immunoblotting with an anti-GFP antibody. The strain used was DY485. (C) DNA damage sensitivity caused by *crb2-2AQ* mutation can be fully rescued by fusing Crb2 with Chk1 kinase. Spot assay was performed as in Figure 2B. Strains used were DY6508, DY6509, DY809, DY6507, DY6510 and DY6511.

As T73 and S80 on Crb2 are critical for Chk1 recruitment to DSBs, we hypothesized that their phosphorylation may facilitate a direct interaction between Crb2 and Chk1. To test this possibility, Crb2(67–85), a 19-amino-acid peptide containing the two functionally important SQ/TQ motifs, together with its mono- and di-phosphorylated forms were synthesized and used in in vitro Chk1 pull-down assays (the amino acid sequence of the peptide is as depicted in Figure 2A). Full-length Chk1 cannot be easily expressed in bacterial cells [34]. Therefore, as input, we used YFP-Flag-His6 (YFH)-tagged Chk1 affinity-purified from *S. pombe* cells with anti-Flag beads [35]. By Coomassie staining of proteins in a polyacrylamide gel, we found that more than 5% of the Chk1 in the input was pulled down by the di-phosphorylated peptide, Crb2(67–85)pT73pS80 (Figure 4A). The peptide mono-phosphorylated at T73, Crb2(67–85)pT73, pulled down slightly lower amount of Chk1 than the di-phosphorylated peptide. Crb2(67–85)pS80 showed significantly weaker affinity but still pulled down clearly visible amount of Chk1. In contrast, unphosphorylated Crb2(67–85) did not pull down a detectable amount of Chk1. As Chk1 was the only dominant band in the phosphopeptide pull-down lanes on the Coomassie-stained gel, we surmise that Chk1 probably bound to the phosphopeptides directly, and if there were other proteins bridging the interactions, they had to act in a highly substoichiometric manner. The abilities of both mono- and di-phosphorylated forms of Crb2 peptides to bind Chk1 in vitro are consistent with the data that mutating either T73 or S80 only partially affected Chk1 activation in vivo. To better quantitate the affinity difference between the phosphorylated and unphosphorylated peptides, we repeated the pull-down assay and used the more sensitive immunoblotting method to estimate the levels of peptide-bound Chk1 (Figure 4B). Using serial dilutions of input as standards, we determined that Crb2(67–85)pT73pS80 and Crb2(67–85)pT73 pulled down about 7% of the input, whereas Crb2(67–85)pS80 pulled down about 1% of the input. Again, we were not able to detect any Chk1 signal in the eluate from the unphosphorylated peptide, but could only estimate that if there was any Chk1, the amount had to be lower than 0.08% of the input (Figure 4B). The phosphopeptide binding by Chk1 not only requires a phosphate group on the peptide but also is sequence context dependent, as a phosphorylated histone H2A peptide can pull down Crb2 but not Chk1 (Figure S7). Together, these results suggest that phosphorylation of the SQ/TQ cluster on Crb2 promotes a direct and specific interaction between Crb2 and Chk1.

### Fusing Crb2 with Chk1 bypasses the requirement for the SQ/TQ cluster

If the only defect of Crb2-2AQ mutant is its inability to engage a physical interaction with Chk1, we predicted that by artificially tethering Crb2 and Chk1 together, we might be able to rescue this defect. To test this possibility, we constructed a strain expressing a Chk1-Crb2 fusion protein as the only version of Crb2 in the cells. This strain was nearly as resistant as wild type to a wide range of genotoxins, suggesting that fusing Crb2 with Chk1 did not significantly attenuate Crb2 functions or otherwise grossly perturb checkpoint signaling (Figure 4C). Remarkably, in the same genetic background, when we mutated both T73 and S80, the resulting strain, *chk1-crb2-2AQ*, behaved exactly like the strain expressing the wild-type fusion protein (Figure 4C), indicating that the defect caused by the 2AQ mutations was completely rescued by the enforced interaction between Crb2 and Chk1. Together with the in vitro binding data, these results suggest that the only essential role of the Crb2 SQ/TQ cluster is to promote a phosphorylation-dependent interaction between Crb2 and Chk1.

### Targeting the Crb2(67–85) peptide to DSBs allows Chk1 focus formation in the absence of endogenous Crb2

It has been shown in mammalian cells that checkpoint effector kinases Chk2 and Chk1 are phosphorylated and activated at sites of DNA damage [36]–[38]. Thus, a parsimonious model for the action of a checkpoint mediator like Crb2 calls for two, and only two, essential functions: first, it needs to recognize the DNA lesions by binding to DNA damage sensors or other upstream signaling components; second, it should be able to interact with the downstream effector kinase and bring it to sites of DNA damage. Such a model has not been formally demonstrated for any checkpoint mediators because it is not yet clear whether these two functions are imparted by separable parts of a mediator. Our previous study has established that Crb2 relocalization to DSBs requires sequence features outside of the SQ/TQ cluster, such as the T215 residue and the C-terminal histone-binding domains [21]. Here we show that the Crb2 SQ/TQ cluster is dispensable for Crb2 relocalization, but is essential for the Crb2-Chk1 interaction. Thus, we postulated that Crb2 may conform to a modular organization and has domains separately responsible for the DSB targeting function and the effector recruitment function. As the Crb2(67–85) phosphopeptide is sufficient for Chk1 binding in vitro, we envisioned that by artificially tethering this peptide to DSBs, where the Rad3-mediated phosphorylation of this peptide presumably can happen, we may be able to bypass the need for the remaining portion of Crb2 that provides the DSB targeting function. To test this hypothesis, we fused Crb2(67–85) to Rad22 protein.

**Figure 5:**
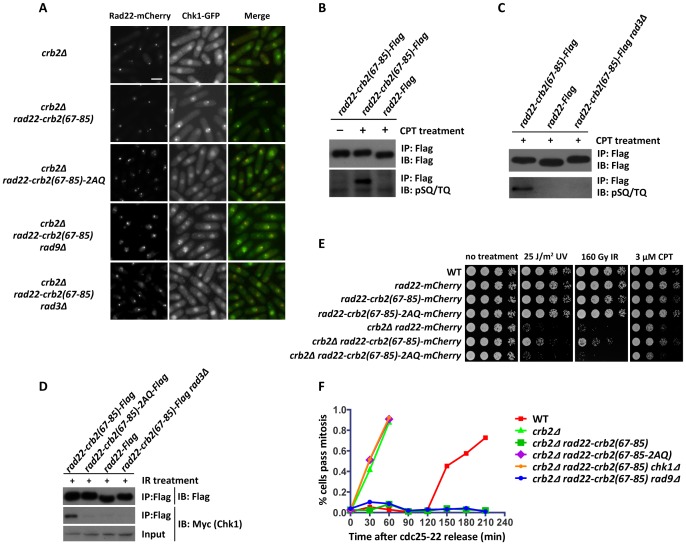
Fusing the Crb2(67–85) peptide with homologous recombination protein Rad22 can bypass Crb2 and Rad9 for Chk1 recruitment and activation. (A) Rad22-Crb2(67–85) can recruit Chk1 to DSBs in the absence of Crb2 and Rad9, but not when the SQ/TQ motifs are mutated, or in the absence of Rad3. Cells were treated with 80 Gy IR and examined by fluorescence microscopy after 1.5 h. Strains used were DY6536, DY6534, DY6535, DY6989 and DY6538. Bar, 5 µm. (B and C) The Crb2(67–85) peptide fused with Rad22 is phosphorylated by Rad3 upon DNA damage treatment. Cells expressing Flag-tagged Rad22 or Flag-tagged Rad22-Crb2(67–85) fusion protein were untreated or treated with 20 µM CPT for 2 h. Rad22 was immunoprecipitated using anti-Flag beads, eluted by boiling in SDS loading buffer, and immunoblotted with anti-Flag or anti-phospho-SQ/TQ antibody. Strains used were DY6561, DY6562 and DY6565. (D) The Crb2(67–85) peptide fused with Rad22 interacts with Chk1 in a manner dependent on Rad3 kinase and the SQ/TQ motifs. Cells expressing Myc-tagged Chk1 were treated with 320 Gy IR. Rad22 was immunoprecipitated using anti-Flag beads, and Chk1 was detected by immunoblotting with anti-Myc antibody. Strains used were DY6561, DY6562, DY6563 and DY6565. (E) DNA damage sensitivity of *crb2Δ* can be partially rescued by expressing a Rad22-Crb2(67–85) fusion protein. Spot assay was performed as in Figure 2B. Strains used were DY6539, DY6540, DY6541, DY6543, DY6536, DY6534 and DY6535. (F) Rad22-Crb2(67–85) can rescue the checkpoint defect of *crb2Δ* in a manner dependent on the SQ/TQ motifs and Chk1, but not Rad9. The indicated mutants in a *cdc25-22* background were synchronized at late G2 phase by incubating at 35.5°C for 2.5 h. Following 80 Gy IR treatment, cultures were returned to the permissive temperature of 25°C. Mitosis was monitored by staining cells with Hoechst and Calcofluor dyes. Strains used were DY6551, DY6550, DY6552, DY6553, DY6555 and DY6554.

As a DNA repair protein recruited to DSBs independently of checkpoint factors, Rad22 can form IR-triggered foci in *crb2Δ* cells (Figure 5A). Chk1 distribution after IR treatment was monitored in *crb2Δ* cells expressing the Rad22 fusion protein. Remarkably, in *crb2Δ rad22-crb2(67–85)* cells, Chk1-GFP formed distinct nuclear foci, which were colocalized with the foci formed by the mCherry-tagged Rad22 fusion protein. In fact, the Chk1 foci in these cells were much brighter than the foci we saw in similarly IR-treated wild-type cells, probably due to the higher local concentration of Rad22 protein at DSBs than that of Crb2. In contrast, *crb2Δ rad22-crb2(67–85)-2AQ* cells behaved exactly like *crb2Δ* in that no Chk1 foci were detected (Figure 5A). Thus, the SQ/TQ cluster represented by the 19-amino-acid peptide Crb2(67–85), when targeted to DSBs, is sufficient for mediating Chk1 relocalization in a manner that maintains the need for the two SQ/TQ motifs, presumably due to the phosphorylation-dependent nature of the Crb2-Chk1 interaction.

### Crb2(67–85) fused to Rad22 protein is phosphorylated at the SQ/TQ motifs upon DNA damage and can be co-immunoprecipitated with Chk1

Even though we were not able to detect SQ/TQ cluster phosphorylation on endogenous Crb2 using phospho-specific antibodies, the strong Chk1 foci in *rad22-crb2(67–85)* cells prompted us to attempt this approach again on the SQ/TQ cluster peptide fused to Rad22. In an immunoblot analysis, a commercially available anti-pSQ/TQ antibody reacted with Rad22-Crb2(67–85) immunoprecipitated from cells treated with CPT, but did not react with Rad22-Crb2(67–85) from untreated cells, nor with Rad22 alone (Figure 5B), suggesting that T73 and/or S80 residues were phosphorylated in response to DNA damage.

The DNA damage-inducible nature of the SQ/TQ cluster phosphorylation is consistent with our preposition that T73 and S80 are substrate sites of Rad3 kinase, the only ATM/ATR family kinase essential for checkpoint signaling in fission yeast. To further verify this hypothesis, we examined the phosphorylation of Rad22-Crb2(67–85) in *rad3Δ* mutant. As predicted, the phosphorylation-specific immunoblot signal was abolished in *rad3Δ* cells (Figure 5C). Another prediction we can make is that Rad3 should be required for Rad22 fusion-mediated Chk1 accumulation at DSBs. Indeed, we found that *rad3Δ* abolished Chk1 foci in *crb2Δ rad22-crb2(67–85)* cells (Figure 5A).

We and others have not been able to detect the physical interactions between endogenous Chk1 and Crb2, most likely due to the transient nature of the interactions [26]. However, in accordance with the strong Chk1-GFP foci we observed in *rad22-crb2(67–85)* cells, we found that Chk1 could be co-immunoprecipitated with Flag-tagged Rad22-Crb2(67–85), in a manner dependent on the SQ/TQ motifs and Rad3 kinase (Figure 5D).

### Rad22-Crb2(67–85) partially rescues the DNA damage sensitivity of *crb2Δ* and is sufficient for a checkpoint arrest

To assess the functional consequences of Chk1 relocalization mediated by Rad22-Crb2(67–85), we analyzed the DNA damage sensitivity of cells expressing Rad22-Crb2(67–85). In *crb2^+^* background, expressing this fusion protein as the only version of Rad22 did not significantly enhance the sensitivity, suggesting that the DNA repair function of Rad22 was not grossly compromised by the fusion (Figure 5E). In *crb2Δ* background, cells expressing Rad22-Crb2(67–85) showed stronger resistance to UV, IR, and CPT treatment compared to cells expressing Rad22 not fused with Crb2 peptide. We note that this rescuing effect was incomplete, as the cells were still more sensitive than the *crb2^+^* strain. This partial rescue requires the SQ/TQ motifs, as the *crb2Δ* cells expressing Rad22-Crb2(67–85)-2AQ did not show improved genotoxin resistance (Figure 5E).

*crb2Δ* cells expressing Rad22-Crb2(67–85) appeared to be capable of checkpoint arrest as they became significantly elongated after DNA damage treatment (Figure 5A). To more directly monitor checkpoint arrest, we performed a *cdc25-22* block-and-release assay. Cells synchronized in G2 by the temperature-sensitive *cdc25-22* mutation were irradiated with IR and then released to permissive temperature to allow mitotic entry. *crb2Δ* cells rapidly entered mitosis after the release, whereas wild type cells showed a checkpoint response as their mitotic entry was delayed for 2 h compared to *crb2Δ* cells (Figure 5F). Strikingly, *crb2Δ* cells expressing Rad22-Crb2(67–85) did not enter mitosis during the observation period of more than 3 h, suggesting that they were capable of a robust checkpoint arrest. The prolonged arrest could be due to slower DNA repair, or defective checkpoint recovery, or a combination of both. This checkpoint arrest is entirely dependent on Chk1, as *chk1Δ* completely abolished the mitotic delay (Figure 5F). Mutating the two SQ/TQ motifs in the Rad22 fusion protein also rendered the cells completely defective in checkpoint arrest (Figure 5F). Together, our observations suggest that artificially tethering Crb2(67–85) to DSBs by a Rad22 fusion is sufficient for recruiting Chk1 to DSBs and activating a Chk1-dependent checkpoint response in the absence of endogenous Crb2.

### Rad22-Crb2(67–85) bypasses the need for 9-1-1 complex in Chk1 relocalization and activation

The PCNA clamp-like 9-1-1 complex, composed of Rad9, Rad1, and Hus1, and its clamp loader, the Rad17-RFC complex, are essential for DNA damage-induced Chk1 phosphorylation and activation by Rad3 [5]. The roles of 9-1-1 complex in Chk1 activation are not entirely clear. On one hand, it may act as a recruitment platform for downstream factors at DSBs by interacting with Rad4/Cut5 [24], which in turn binds to Crb2 [11], [21], and eventually brings Chk1 to the proximity of Rad3 at DSBs through the Crb2-Chk1 interaction we report here. Consistent with this model, we found that Chk1 focus formation requires Rad9 (Figure S2), and deletion of Rad9 or Rad17 abolished Rad4/Cut5 accumulation at DSBs (Figure S8). However, on the other hand, it has also been shown that the orthologs of Rad9 and Rad4/Cut5 in budding yeast and the ortholog of Rad4/Cut5 in vertebrates possess the so-called ATR-activating domains, which are sufficient for activating ATR kinase in vitro and important for checkpoint activation in vivo [39]–[45]. Thus, it is possible that in fission yeast, 9-1-1 complex enhances Rad3 activity either directly or indirectly through recruiting Rad4/Cut5.

Our finding that artificially tethering a Crb2(67–85) peptide to DSBs is sufficient for Chk1 relocalization and activation provides a means to assess the functional importance of the two proposed roles of 9-1-1 in checkpoint signaling. We anticipated that Rad22-Crb2(67–85) may be able to bypass the Crb2-recruitment role of 9-1-1. However, if the putative ATR-activating role of Rad9 and Rad4/Cut5 is essential for the phosphorylation of either Crb2 SQ/TQ cluster or Chk1 by Rad3, Rad22-Crb2(67–85) would not be sufficient for Chk1 activation in the absence of 9-1-1.

As a readout for Rad3-dependent phosphorylation of Crb2 SQ/TQ cluster, we examined Chk1 localization in *rad9Δ crb2Δ* cells expressing Rad22-Crb2(67–85). Remarkably, Chk1 formed nuclear foci colocalizing with the Rad22 fusion protein (Figure 5A), suggesting that Rad3 is able to phosphorylate Crb2 SQ/TQ cluster in the absence of 9-1-1 if we bypass the recruitment function of 9-1-1. We went on to test whether checkpoint can be activated under such a circumstance. We found that, in a *cdc25-22* block-and-release experiment, *rad9Δ crb2Δ rad22-crb2(67–85)* cells arrested cell cycle progression in response to IR treatment (Figure 5F). This result indicates that abolishing 9-1-1 functions did not block checkpoint activation when Chk1 is recruited to DSBs by Rad22-Crb2(67–85). Thus, the main DNA damage checkpoint function of 9-1-1 appears to be recruiting Crb2 and, in turn, Chk1.

### Tethering Crb2(67–85) to Rad4/Cut5 completely rescues the checkpoint defect and DNA damage sensitivity of *crb2Δ*

Rad22-Crb2(67–85) fusion did not fully rescue *crb2Δ*, possibly due to the differences in DSB recruitment kinetics and local microenvironment compared to native Crb2. As Rad4/Cut5 recruits Crb2 to DSBs, we reasoned that fusing Crb2(67–85) to Rad4/Cut5 may allow a better bypass of endogenous Crb2.

**Figure 6:**
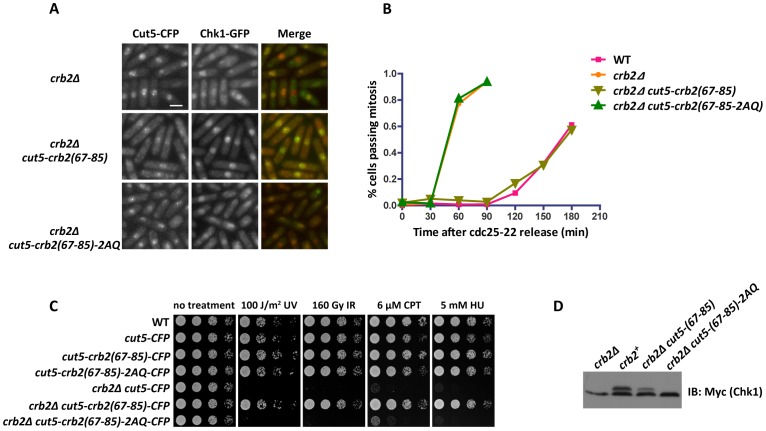
Fusing the Crb2(67–85) peptide with Rad4/Cut5 is sufficient for Chk1 recruitment and activation in the absence of endogenous Crb2. (A) Fusing Crb2(67–85) with Rad4/Cut5 bypasses Crb2 for Chk1 focus formation. Cells were challenged with S-phase IR treatment as in Figure 1A and then examined by fluorescence microscopy. Strains used were DY6512, DY6513 and DY6514. Bar, 5 µm. (B) Fusing Crb2(67–85) with Rad4/Cut5 bypasses Crb2 for checkpoint activation. The *cdc25-22* block-and-release assay was performed as in Figure 5F. Strains used were DY6546, DY6547, DY6548 and DY6549. (C) DNA damage sensitivity of *crb2Δ* is fully rescued by Cut5-Crb2(67–85) fusion. Spot assay was performed as in Figure 2B. Strains used were DY6517, DY6518, DY6519, DY6520, DY6512, DY6513 and DY6514. (D) Chk1 phosphorylation defect of *crb2Δ* is rescued by Cut5-Crb2(67–85) fusion. Cells were treated with 20 µM CPT for 2 h. Cell lysates were separated on SDS-PAGE and probed with anti-Myc antibody. Strains used were DY6521, DY6522, DY6523 and DY6524.

As we expected, expressing Cut5-Crb2(67–85) in *crb2Δ* cells rescued the Chk1 focus formation defect and restored the checkpoint signaling as the cells arrested in a mononucleated, elongated state after DNA damage treatment (Figure 6A). In contrast, *crb2Δ cut5-crb2(67–85)-2AQ* cells behaved exactly like *crb2Δ* in that no Chk1 foci were detected and the cells failed to arrest in response to DNA damage (Figure 6A). In a *cdc25-22* block-and-release assay, we found that *crb2Δ cut5-crb2(67–85)* cells delayed mitosis after IR treatment to the same extent as wild type, whereas *crb2Δ cut5-crb2(67–85)-2AQ* cells resumed cell cycle progression as quickly as *crb2Δ* cells (Figure 6B). Consistent with the rescuing of the checkpoint defect, hypersensitivities of *crb2Δ* to several genotoxins were fully rescued by expressing Cut5-Crb2(67–85) (Figure 6C). Furthermore, Chk1 phosphorylation defect of *crb2Δ* was substantially alleviated by expressing Cut5-Crb2(67–85) (Figure 6D). Together, these data suggest that the DSB targeting function fulfilled by Crb2 sequence outside of amino acids 67–85 can be completely bypassed by a Rad4/Cut5 fusion, and the Crb2(67–85) peptide, when properly targeted to DSBs, can carry out all the checkpoint functions of full-length Crb2.

## Discussion

**Figure 7:**
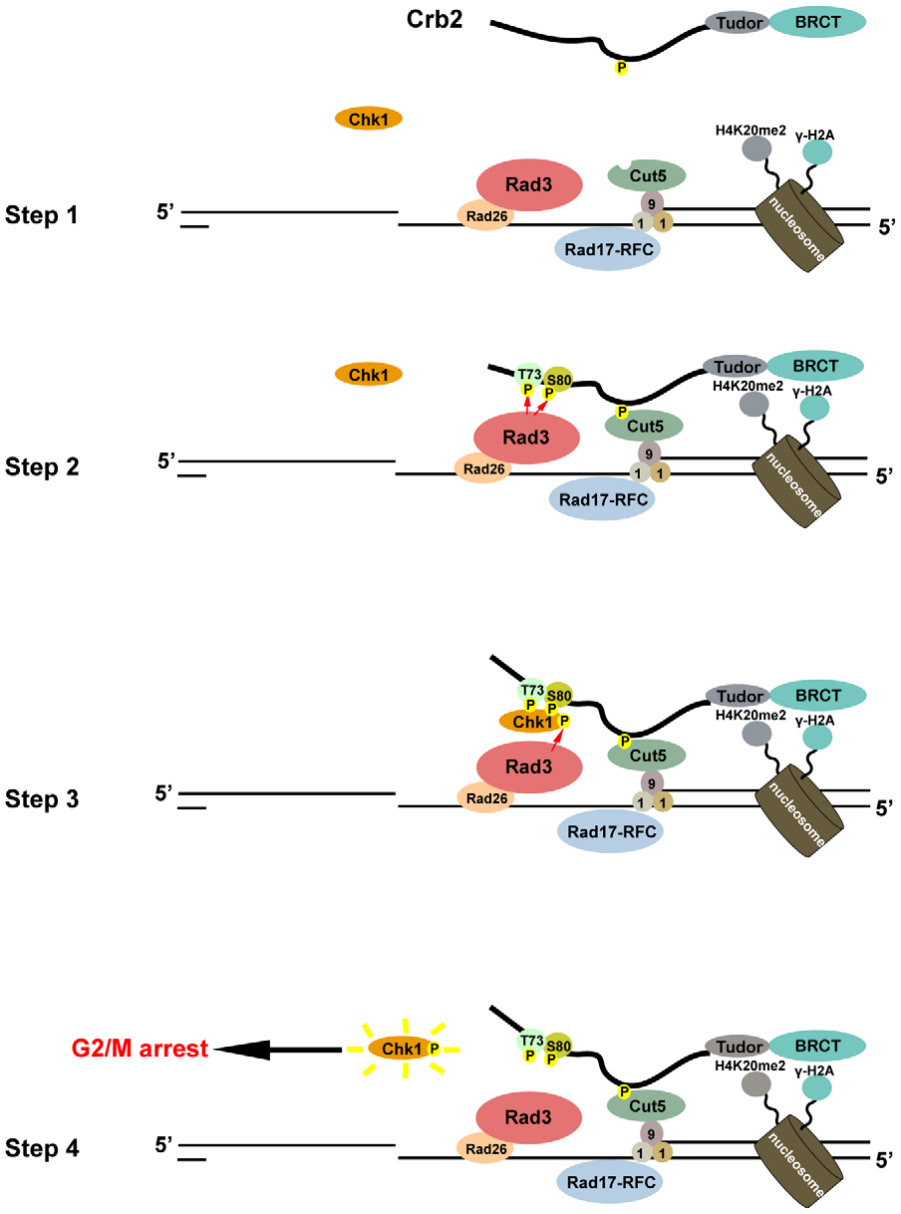
A model of how Crb2 mediates Chk1 activation. In step 1, DSB formation induces the phosphorylation of H2A (**γ**-H2A) on surrounding chromatin. Upon DSB resection, Rad3 and 9-1-1 are recruited to single-stranded DNA and single-strand/double-strand junction, respectively. Rad4/Cut5 is also recruited via binding to Rad9. In step 2, through its interactions with modified histones and Rad4/Cut5, Crb2 relocalizes to the DSB and becomes phosphorylated at the SQ/TQ cluster by Rad3. In step 3, phosphorylated SQ/TQ cluster interacts with Chk1 and promotes its phosphorylation by Rad3. In step 4, the activated Chk1 molecule leaves the DSB to fulfill its effector function and allows further rounds of Chk1 activation to occur.

In this study, we found that a pair of SQ/TQ motifs in the N-terminal region of Crb2 is critical for its checkpoint mediator function. We show that these motifs are likely in vivo target sites for phosphorylation by Rad3 kinase. Remarkably, a 19-amino-acid peptide containing these SQ/TQ motifs is sufficient for mediating a phosphorylation-dependent interaction with Chk1 in vitro and promoting Chk1 activation in vivo when targeted to DSBs. Hence, we conclude that Crb2 uses a phosphorylation-dependent Chk1-binding module to recruit Chk1 to DSBs and thereby allow it to be phosphorylated and activated by Rad3 (Figure 7).

### Crb2 SQ/TQ cluster interacts with Chk1 in a phosphorylation-dependent manner

Multiple lines of evidence suggest that T73 and S80 residues in Crb2 are phosphorylated in response to DNA damage. First, the DNA damage-induced Crb2 mobility shift was significantly diminished by 2AQ mutations in both wild-type and 8AQ mutant context. Second, anti-phospho-SQ/TQ antibody specifically recognized the Crb2(67–85) peptide fused to Rad22 after DNA damage in a Rad3-dependent manner. Third, the requirement of these residues for the co-immunoprecipitation of Rad22-Crb2(67–85) and Chk1, and the rescue of the 2AQ mutations by a Chk1-Crb2 fusion strongly suggest that these residues mediate a Crb2-Chk1 interaction in vivo, and correspondingly, the in vitro interaction between the Crb2(67–85) peptide and Chk1 requires the phosphorylation of at least one of these residues. Fourth, mass spectrometry analysis showed that the S80 residue is phosphorylated in vivo. Even though we did not obtain direct evidence that T73 residue is phosphorylated in vivo, there are good reasons to believe this is the case. First, T73 is in a conserved LxLTQLFE motif, which fits the preference of ATR kinase for hydrophobic residues at the −1 and −3 positions of it substrate sites [46]. Second, the Crb2(67–85) peptide singly phosphorylated at the T73 residue showed robust binding to Chk1 in vitro. To obtain additional corroborating evidence, we have attempted to create phospho-mimetic mutants, but substituting both of these residues to either glutamate or aspartate resulted in the same phenotypes as the 2AQ mutant (our unpublished observations), suggesting that proper checkpoint mediator function of Crb2 needs phosphorylation and not simply negatively charged side chains at these positions.

Neither T73A nor S80A mutation alone strongly affected the checkpoint mediator function of Crb2, whereas the 2AQ mutations completely abolished Chk1 recruitment and activation, indicating that these two phosphorylation sites play redundant roles. Correspondingly, the Crb2(67–85) peptide phosphorylated on either T73 or S80 is able to pull down Chk1. The weaker in vitro binding affinity of S80-phosphorylated peptide suggests that once the binding strength is above a certain minimal threshold, Crb2 is able to fulfill its role in recruiting Chk1 to DSBs. Alternatively, our in vitro binding assay conditions may have not faithfully mimicked the in vivo environment and underestimated the true Chk1-binding ability of S80-phosphorylated Crb2.

The conservation of Crb2 SQ/TQ cluster may not be restricted to the fission yeast species. A pair of neighboring SQ/TQ sites in a similar sequence context also exists in Crb2 orthologs in many other *Ascomycota* fungi species, such as *Neurospora crassa* and *Aspergillus nidulans* (Figure S9), suggesting that the mechanism we describe here may represent an ancient and conserved mode of Chk1 activation by its mediator. We failed to detect similar sequence motifs in budding yeast scRad9, and a previous study had assigned the Chk1 activation function to the 40–200 amino acid region of scRad9, which does not contain any SQ/TQ sites [13]. Thus, scRad9 may have evolved a different way of binding to and activating Chk1, or alternatively, the ATR-like Mec1 kinase may phosphorylate the 40–200 amino acid region of scRad9 on non-SQ/TQ sites, as has been shown for the Mec1-mediated phosphorylation of Rad53 [47].

In metazoans, Claspin mediates the activation of Chk1 [14], [48]. It has been suggested that Claspin is related by sequence homology to the replication checkpoint mediator Mrc1 in yeasts [8], [9]. Thus, it is unlikely that Claspin and Crb2 share evolutionary ancestry. Despite this, our findings have revealed mechanistic similarities between the ways Claspin and Crb2 mediate Chk1 activation, namely, both Claspin and Crb2 undergo ATR/Rad3-dependent phosphorylation on multiple sites, and these phosphorylation events promote interactions with Chk1 kinase [28], [30]. There is also a notable difference. The Chk1-binding region in Crb2 is phosphorylated on SQ/TQ motifs, probably by Rad3, whereas the phosphorylation sites in the Chk1-binding region of Claspin are SG motifs directly phosphorylated by casein kinase 1 gamma 1 [31].

The Chk2 family effector kinases harbor one or two FHA domains, which are phosphopeptide-binding modules and can interact directly with their respective checkpoint mediators in a phosphorylation-dependent manner [10], [49]–[52]. In contrast, Chk1 family kinases do not have any known phosphopeptide-binding domain. There are two conserved domains in Chk1, the N-terminal kinase domain and the C-terminal regulatory domain. Vertebrate Chk1 appears to use its kinase domain to interact with phosphorylated Claspin [27]. However, in *S. cerevisiae*, conserved sequence motifs in the C-terminal domain of Chk1 were shown to be required for a yeast two-hybrid interaction between Chk1 and scRad9 [53]. We have attempted to use Crb2 peptide pull-down to identify the region of Chk1 involved in Crb2-Chk1 interaction. Neither the kinase domain nor the C-terminal domain is sufficient for binding with a phosphorylated Crb2(67–85) peptide (our unpublished observations), suggesting that both domains of Chk1 contribute to Crb2-Chk1 interaction.

### Crb2 mediates Chk1 activation by recruiting it to sites of DNA damage

Previous studies have established the functional importance of phosphorylation-dependent interactions for the mediator-effector pairs of Claspin-Chk1 in vertebrates [28], [30], scRad9-Rad53 in budding yeast [51], and Mrc1-Cds1 in fission yeast [10], [52]. We show here that an SQ/TQ cluster-mediated Crb2-Chk1 interaction is critical for Chk1 activation in fission yeast. Thus, it appears that a common feature of the checkpoint signaling pathways is an essential direct interaction between checkpoint mediator and its downstream effector kinase. How such an interaction facilitates the activation of effector kinase is not entirely clear. As the ATR-mediated phosphorylation of aforementioned effector kinases is necessary for their activation, a current consensus of the field is that mediator-effector interactions increase the efficiency of ATR-catalyzed phosphorylation of effectors [10], [29], [47]. Two models, not exclusive to each other, can account for the impact of mediator-effector interactions on effector phosphorylation. The first model postulates that mediators directly participate in the phosphorylation reactions, either by increasing the enzyme-substrate affinity through simultaneously interacting with ATR and the effector, or by alternating the conformation of the effector to make it a better substrate for ATR kinase. Evidence supporting this model came from cell-free or reconstituted in vitro systems, showing that the presence of a mediator boosts the phosphorylation of its corresponding effector but not a generic ATR substrate [29], [47], [54], [55]. As the spatial organization of cellular components was not maintained in these in vitro systems, the roles of spatial regulation could not be assessed. The other model suggests that a DNA damage-induced mediator-effector interaction alters the spatial distribution of an effector and brings it to DNA lesions, where ATR kinase also accumulates. As a consequence, the effector phosphorylation is enhanced due to heightened local concentrations of both enzyme and substrate. Consistent with this model is the fact that all mediators are capable of relocalizing to the proximity of aberrant DNA structures that trigger checkpoint signaling. For example, the DNA damage checkpoint mediators Crb2 and scRad9 form nuclear foci at DSBs [15], [56], and the replication checkpoint mediators Mrc1 and Claspin can bind to branched DNA structures in vitro, which may form at stalled replication forks in vivo [57], [58]. In the case of Crb2, the ability to relocalize to sites of DNA damage is essential for its checkpoint mediator function [21].

Our data here lend further support to the second model, as the fusion of Crb2(67–85) peptide to either Rad22 or Rad4/Cut5 can largely bypass the need of the remaining portion of Crb2 for Chk1 activation. It is unlikely that the same peptide can simultaneously bind to Chk1 and Rad3. Furthermore, as the SQ/TQ motifs, or even the 19-amino-acid Crb2(67–85) peptide sequence, became dispensable when Crb2 and Chk1 were fused together (Figure 4C and Figure S10), the function of this peptide probably does not include altering Chk1 conformation or otherwise directly participating in the phosphorylation of Chk1 by Rad3. Thus, the main checkpoint function of Crb2 appears to be recruiting Chk1 to DNA lesions where Rad3 also resides.

### The 9-1-1 complex is not required for Rad3 activities towards Crb2 and Chk1

In fission yeast, Rad3-mediated phosphorylation of the T412 residue on the Rad9 subunit of the 9-1-1 complex is essential for DNA damage-induced Chk1 activation [24]. This phosphorylation event promotes an interaction between Rad9 and the second pair of BRCT domains in Rad4/Cut5. Consistent with these data, we show here that the formation of Rad4/Cut5 foci at DSBs requires Rad9 (Figure S8). Furthermore, the N-terminal tandem BRCT domains of Rad4/Cut5 mediate an interaction between Rad4/Cut5 and Crb2 [11], [21]. Thus, a recruitment cascade composed of Rad9, Rad4/Cut5, and Crb2 can be envisioned, which eventually leads to the targeting of Crb2 to DSBs (Figure 7). A similar set of phosphorylation-dependent binary interactions between the orthologs of these proteins in budding yeast have been described, suggesting that such a checkpoint mediator recruitment pathway may be conserved [45], [59], [60].

Vertebrate and budding yeast homologs of Rad4/Cut5, as well as the budding yeast homolog of Rad9, possess in vitro ATR-activating activities. Such activities require sequence motifs containing a critical aromatic amino acid. Similar motifs have been found in fission yeast Rad4/Cut5 and Rad9 [43], [44]. Whether Rad4/Cut5 and Rad9 are capable of activating Rad3 awaits verification by biochemical assays. However, we show here that Rad22-Crb2(67–85) fusion can mediate checkpoint signaling in the absence of Rad9. Thus, the Rad3 kinase activities towards Crb2(67–85) and Chk1 do not absolutely need 9-1-1, and the main function of 9-1-1 complex during DNA damage checkpoint signaling appears to be recruiting Crb2 to DSBs. This conclusion is consistent with recent reports showing that abolishing the ATR-activating activities of both ATR activators in budding yeast does not cause as strong defects as the loss of ATR ortholog Mec1 [44], [45]. It is also consistent with studies showing that activation of Chk1 by Tel1 (ATM), as revealed in strains lacking Ctp1 DSB resection factor, also requires the 9-1-1 complex [4].

## Materials and Methods

### Yeast strains

Strains used in this study are listed in Table S1.

### Microscopy for monitoring nuclear focus formation

Cells were maintained in logarithmic phase in EMM minimal media at 30°C. Microscopy was performed using a DeltaVision personalDV system equipped with a CFP/YFP/mCherry filter set (Chroma 89006 set) and a Photometrics CoolSNAP HQ2 camera. Images were acquired with a 100×, 1.4-NA objective. Four Z-sections at 0.5-µm intervals were merged into one image using the maximum intensity projection method with the softWoRx software.

### Crb2 and Chk1 mobility shift assay

Whole cell extracts were prepared by boiling 10 OD_600_ units of cells with 100 µl SDS loading buffer following a 0.35 M NaOH treatment. To assess the mobility shift of Myc-tagged Chk1, samples were run on 10% SDS-PAGE (Bis-acrylamide∶acrylamide ratio of 1∶100) and immunoblotted with a polyclonal anti-Myc antibody (Santa Cruz, sc-789). To detect Crb2 mobility shift, samples were run on 6% SDS-PAGE and immunoblotted with a polyclonal anti-Crb2 antibody (Du et al., 2003).

### Chk1 purification and Crb2(67–85) peptide pull down

Proteins were extracted from about 1000 OD_600_ units of cells by glass bead beating (FastPrep-24) in lysis buffer (50 mM Hepes, pH 7.5, 100 mM NaCl, 1 mM EDTA, 10% glycerol, 0.05% NP-40, 1 mM PMSF, 1.5 mM DTT, 1× Protease Inhibitor Cocktail (Roche), 1× PhosSTOP (Roche)). Anti-Flag M2 affinity gel (Sigma, A2220) was applied to immunoprecipitate YFH-tagged Chk1. After binding, beads were briefly washed with lysis buffer and eluted with 3×Flag peptide. Eluted Chk1 was incubated with 2 µg biotin-labeled Crb2(67–85) peptides at 4°C for 2 hours. Then 30 µl pre-washed Dynabeads M-280 Streptavidin (Invitrogen) was added to pull down peptides. Beads were briefly washed with lysis buffer and eluted with SDS loading buffer. Chk1 was detected by Coomassie staining or immunoblotting with an anti-GFP antibody (Roche #11814460001).

### Immunoprecipitation of Flag-tagged Rad22

Protein extraction and Flag-IP were performed as above except eluting from anti-Flag M2 affinity gel by boiling with SDS loading buffer. Immunoblotting was performed with a polyclonal anti-Flag antibody (Sigma, F7425) and an anti-pSQ/TQ antibody (Cell Signaling #2851).

### Mass spectrometry analysis of Crb2 phosphorylation

TAP-tagged Crb2 was purified from IR-treated cells using IgG Sepharose beads and eluted by TEV protease cleavage. The eluate was dissolved in 8 M urea, 100 mM Tris, pH 8.5, reduced with 5 mM TCEP for 20 min, and alkylated with 10 mM iodoacetamide for 15 min in the dark, all at the room temperature. Then the sample was split into three aliquots, digested separately overnight at 37°C with trypsin (in 2 M urea, 1 mM CaCl2, 100 mM Tris, pH 8.5), elastase (in 2 M urea, 100 mM Tris, pH 8.5), or subtilysin (in 6 M urea, 100 mM Tris, pH 8.5). The digestions were stopped with 5% formic acid (final concentration). Peptides from three digestions were combined and loaded onto a desalting column (250-µm i.d. fused silica capillary column with 2 cm Aqua C18 resin (Phenomenex) with a 2-µm filtered union). After desalting, a 100-µm i.d. column packed with 10 cm of Aqua C18 resin and 2 cm of Partisphere SCX resin (Whatman) was connected to the desalting column through the filtered union. MS analysis was performed on LCQ Deca mass spectrometer (Thermo-Finnigan) using a 12-step MudPIT method described previously [33]. The MS/MS spectra were searched with SEQUEST [61] with or without an addition of 80 on S, T, or Y (phosphorylation) against an *S. pombe* protein database. The search results were combined and filtered with DTASelect [62].

## Supporting Information

Figure S1 Chk1-GFP is fully functional and displays a diffuse nuclear distribution in the absence of DNA damage. (A) Cells expressing Chk1-GFP as the only version of Chk1 do not show DNA damage hypersensitivity compared to wild type (WT). Spot assay was performed as in Figure 2B. Strains used were LD346, LD2 and DY6517. (B) Chk1-GFP distribution in the absence of DNA damage. Cells grown to logarithmic phase in EMM medium were examined by fluorescence microscopy. The strain used was DY6498. Bar, 5 µm.
(PDF)

Figure S2 DNA damage-induced Chk1 focus formation requires Rad3 and Rad9. Cells expressing Chk1-GFP in WT, *rad3Δ* or *rad9Δ* deletion background were treated with 160-Gy IR and then examined by fluorescence microscopy. Strains used were DY6498, DY6495, DY6496. Bar, 5 µm.
(PDF)

Figure S3 Crb2 SQ/TQ cluster is important for IR resistance and IR-induced Chk1 activation. (A) Crb2 SQ/TQ cluster mutants are sensitive to IR-induced DNA damage. The indicated mutants were treated with different doses of IR, and then plated on YES medium. Colonies were counted two days later. At 320 Gy, 68% of the wild type cells, 42% of the *crb2-T73A* mutant cells, 55% of the *crb2-S80A* mutant cells, 0.57% of the *crb2-2AQ* mutant cells, 0.094% of the chk1Δ cells, and 0.047% of the crb2Δ cells survived. Strains used were DY377, DY369, DY370, DY371, LD195 and LD346. (B) IR-induced Chk1 phosphorylation is defective in Crb2 SQ/TQ cluster mutants. Cells were untreated or treated with 320 Gy of IR. Cell lysates were separated on SDS-PAGE and probed with an anti-Myc antibody by immunoblotting. Strains used were DY377, LD195, DY369, DY370 and DY371.
(PDF)

Figure S4 Crb2 SQ/TQ cluster is important for IR-induced checkpoint arrest. (A) Crb2 SQ/TQ cluster mutants are defective in IR-induced cell cycle arrest. The indicated mutants in a *cdc25-22* background were synchronized at late G2 phase by incubating at 35.5°C for 2.5 h. Following 80 Gy IR or no DNA damage treatment, cultures were returned to the permissive temperature of 25°C. Mitosis was monitored by staining cells with Hoechst and Calcofluor dyes. Strains used were LD715, DY8362, DY8363, DY8365 and DY8367. (B and C) *crb2-2AQ* cells entering mitosis after DNA damage show the “cut” phenotypes. Cells were challenged with S-phase IR treatment as in Figure 2D, and examined by fluorescence microscopy after Hoechst staining. (B) The wild-type cells were arrested in G2 phase, but the *crb2-2AQ* cells entered mitosis and showed the “cut” phenotypes, where septation occurs without proper segregation of nuclear DNA. Arrows point to cells with their nuclear DNA abnormally positioned at the septum. Bar, 5 µm. (C) The percentages of different types of nuclear morphology among the septum-containing *crb2-2AQ*, *chk1Δ* or *crb2Δ* cells. N, the number of septum-containing cells analyzed. Strains used were DY377, LD195, LD346 and DY371.
(PDF)

Figure S5 DNA damage-induced focus formation by Crb2(1–358)-LZ is not perturbed by SQ/TQ cluster mutations. Cells expressing YFP-Crb2(1–358)-LZ or YFP-Crb2(1–358)-2AQ-LZ were observed by fluorescence microscopy after a 16-h induction of HO endonuclease, which cleaves a specific site in the genome. Strains used were DY295 and DY303. Bar, 5 µm.
(PDF)

Figure S6 *crb2-8AQ* mutant with mutations at all SQ/TQ motifs other than T73, S80, and S666, does not show DNA damage hypersensitivity. Spot assay was performed as in Figure 2B. Strains used were LD195, DY6839, DY6845, DY6844, DY6840, DY6841, DY6842 and DY6843.
(PDF)

Figure S7 Chk1 is unable to bind the phosphorylated H2A (γH2A) peptide. (A) BRCTs in Crb2 can bind the γH2A peptide. Recombinant His-GST-Crb2(521–778) was purified from *E. coli* with Ni-NTA beads and incubated with Hta1(120–132) (H2A) or Hta1(120–132-pS129) (γH2A) peptide. Peptides were pulled down by streptavidin Dynabeads and eluted by boiling in SDS loading buffer. The eluates and input were analyzed by 4%–20% SDS-PAGE followed by Coomassie staining. (B) Chk1 cannot bind the γH2A peptide. The experiment was performed as in Figure 4B.
(PDF)

Figure S8 DNA damage-induced Rad4/Cut5 focus formation requires Rad9 and Rad17. Cells expressing Cut5-YFP or Cut5-CFP in WT, *rad9Δ* or *rad17Δ* background were treated with 160 Gy IR and then examined by fluorescence microscopy. Strains used were DY6557, DY6558, DY6559 and DY6560. Bar, 5 µm.
(PDF)

Figure S9 The SQ/TQ cluster region of Crb2 is conserved in its orthologs in *Ascomycota* fungi species outside of the fission yeast clade.
(PDF)

Figure S10 Crb2(67–85) sequence is dispensable when Crb2 is fused with Chk1. Spot assay was performed as in Figure 2B. Strains used were DY809, DY6507, DY6510, DY6511 and DY8046.
(PDF)

Table S1 Strains used in this study.
(DOC)
