# Supplementary figures and images for: Phosphorylation-Dependent Interactions between Crb2 and Chk1 Are Essential for DNA Damage Checkpoint
Source: PLoS Genet. 2012 Jul 5;8(7):e1002817. doi: 10.1371/journal.pgen.1002817 (PMC3390401; doi:10.1371/journal.pgen.1002817)

Qu et al. Figure S1

**A**

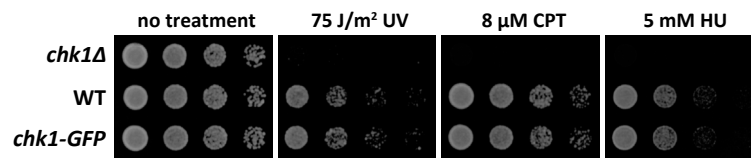

**B**

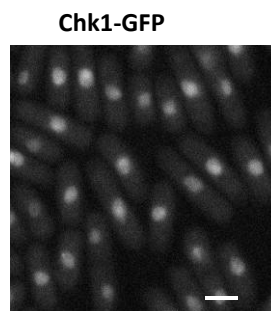

Supplement: Figure S1 — Chk1-GFP is fully functional and displays a diffuse nuclear distribution in the absence of DNA damage. (A) Cells expressing Chk1-GFP as the only version of Chk1 do not show DNA damage hypersensitivity compared to wild type (WT). Spot assay was performed as in Figure 2B. Strains used were LD346, LD2 and DY6517. (B) Chk1-GFP distribution in the absence of DNA damage. Cells grown to logarithmic phase in EMM medium were examined by fluorescence microscopy. The strain used was DY6498. Bar, 5 µm. (PDF) [file pgen.1002817.s001.pdf]

Qu et al. Figure S2

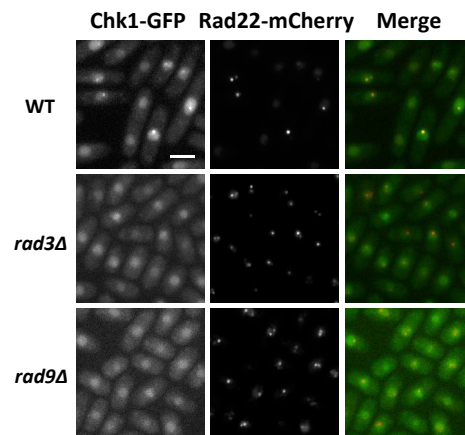

Supplement: Figure S2 — DNA damage-induced Chk1 focus formation requires Rad3 and Rad9. Cells expressing Chk1-GFP in WT, rad3Δ or rad9Δ deletion background were treated with 160-Gy IR and then examined by fluorescence microscopy. Strains used were DY6498, DY6495, DY6496. Bar, 5 µm. (PDF) [file pgen.1002817.s002.pdf]

Qu et al. Figure S3

**A**

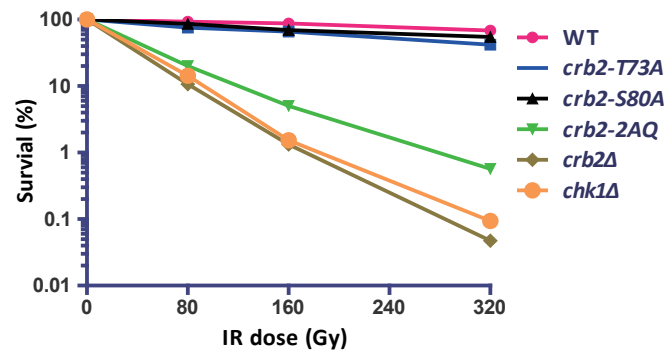

**B**

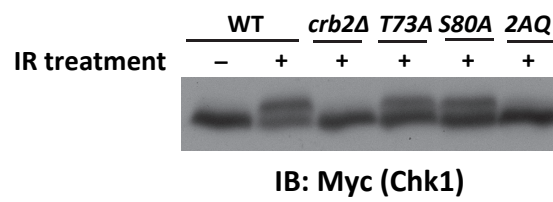

Supplement: Figure S3 — Crb2 SQ/TQ cluster is important for IR resistance and IR-induced Chk1 activation. (A) Crb2 SQ/TQ cluster mutants are sensitive to IR-induced DNA damage. The indicated mutants were treated with different doses of IR, and then plated on YES medium. Colonies were counted two days later. At 320 Gy, 68% of the wild type cells, 42% of the crb2-T73A mutant cells, 55% of the crb2-S80A mutant cells, 0.57% of the crb2-2AQ mutant cells, 0.094% of the chk1Δ cells, and 0.047% of the crb2Δ cells survived. Strains used were DY377, DY369, DY370, DY371, LD195 and LD346. (B) IR-induced Chk1 phosphorylation is defective in Crb2 SQ/TQ cluster mutants. Cells were untreated or treated with 320 Gy of IR. Cell lysates were separated on SDS-PAGE and probed with an anti-Myc antibody by immunoblotting. Strains used were DY377, LD195, DY369, DY370 and DY371. (PDF) [file pgen.1002817.s003.pdf]

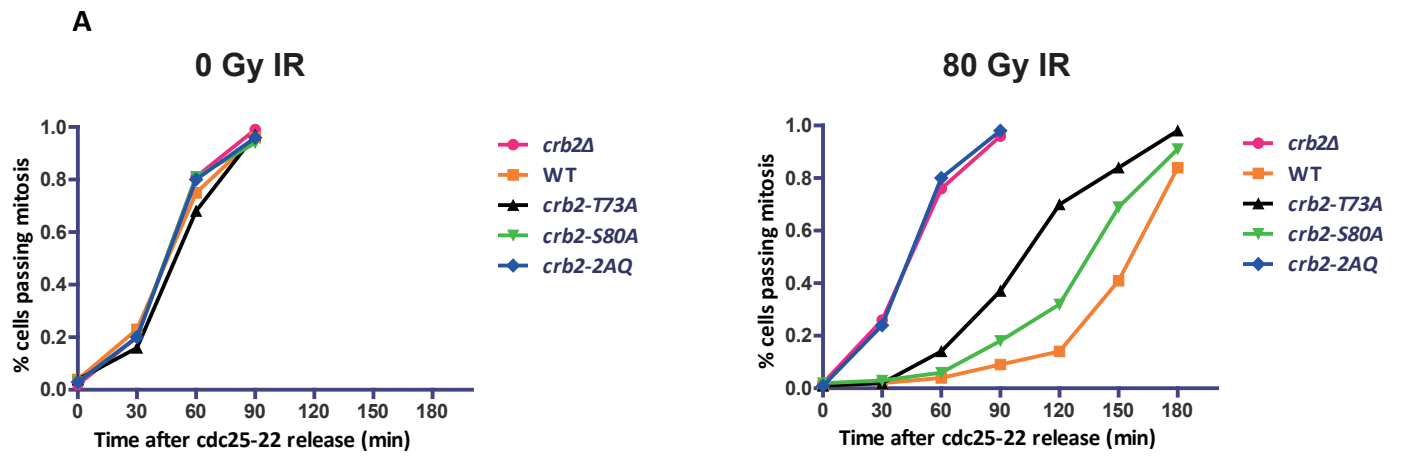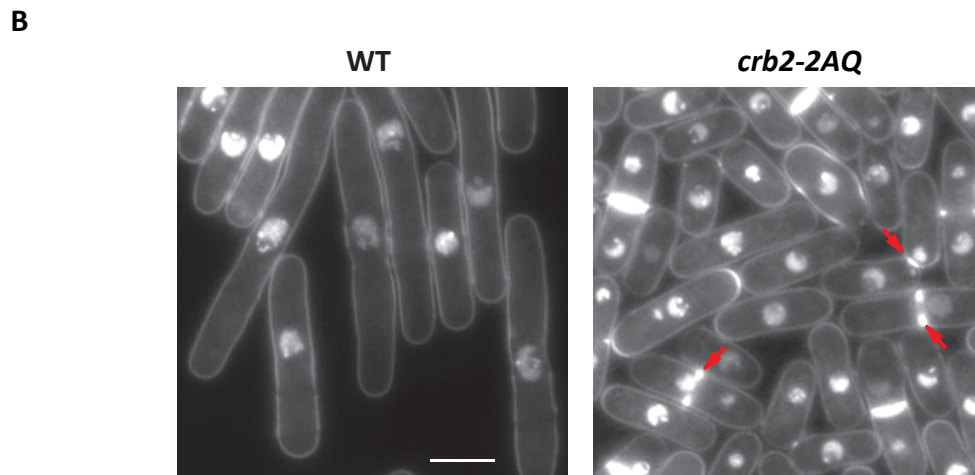

**C**

| <b><i>crb2-2AQ</i><br/>(N=121)</b> | 19% | 47% | 7% | 27% |
|------------------------------------|-----|-----|----|-----|
| <b><i>chk1Δ</i><br/>(N=58)</b>     | 21% | 43% | 7% | 29% |
| <b><i>crb2Δ</i><br/>(N=45)</b>     | 18% | 46% | 4% | 32% |

Supplement: Figure S4 — Crb2 SQ/TQ cluster is important for IR-induced checkpoint arrest. (A) Crb2 SQ/TQ cluster mutants are defective in IR-induced cell cycle arrest. The indicated mutants in a cdc25-22 background were synchronized at late G2 phase by incubating at 35.5°C for 2.5 h. Following 80 Gy IR or no DNA damage treatment, cultures were returned to the permissive temperature of 25°C. Mitosis was monitored by staining cells with Hoechst and Calcofluor dyes. Strains used were LD715, DY8362, DY8363, DY8365 and DY8367. (B and C) crb2-2AQ cells entering mitosis after DNA damage show the “cut” phenotypes. Cells were challenged with S-phase IR treatment as in Figure 2D, and examined by fluorescence microscopy after Hoechst staining. (B) The wild-type cells were arrested in G2 phase, but the crb2-2AQ cells entered mitosis and showed the “cut” phenotypes, where septation occurs without proper segregation of nuclear DNA. Arrows point to cells with their nuclear DNA abnormally positioned at the septum. Bar, 5 µm. (C) The percentages of different types of nuclear morphology among the septum-containing crb2-2AQ, chk1Δ or crb2Δ cells. N, the number of septum-containing cells analyzed. Strains used were DY377, LD195, LD346 and DY371. (PDF) [file pgen.1002817.s004.pdf]

Qu et al. Figure S5

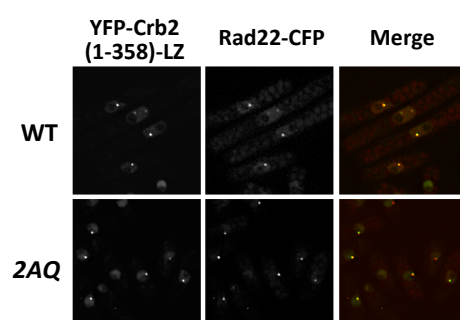

Supplement: Figure S5 — DNA damage-induced focus formation by Crb2(1–358)-LZ is not perturbed by SQ/TQ cluster mutations. Cells expressing YFP-Crb2(1–358)-LZ or YFP-Crb2(1–358)-2AQ-LZ were observed by fluorescence microscopy after a 16-h induction of HO endonuclease, which cleaves a specific site in the genome. Strains used were DY295 and DY303. Bar, 5 µm. (PDF) [file pgen.1002817.s005.pdf]

Qu et al. Figure S6

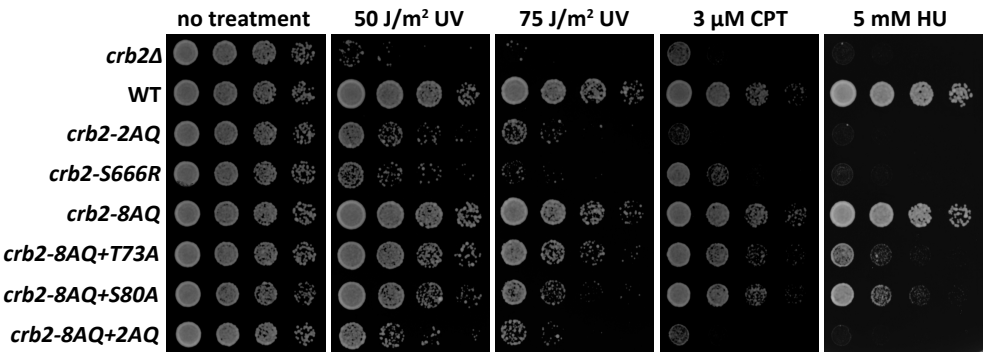

Supplement: Figure S6 — crb2-8AQ mutant with mutations at all SQ/TQ motifs other than T73, S80, and S666, does not show DNA damage hypersensitivity. Spot assay was performed as in Figure 2B. Strains used were LD195, DY6839, DY6845, DY6844, DY6840, DY6841, DY6842 and DY6843. (PDF) [file pgen.1002817.s006.pdf]

**A**

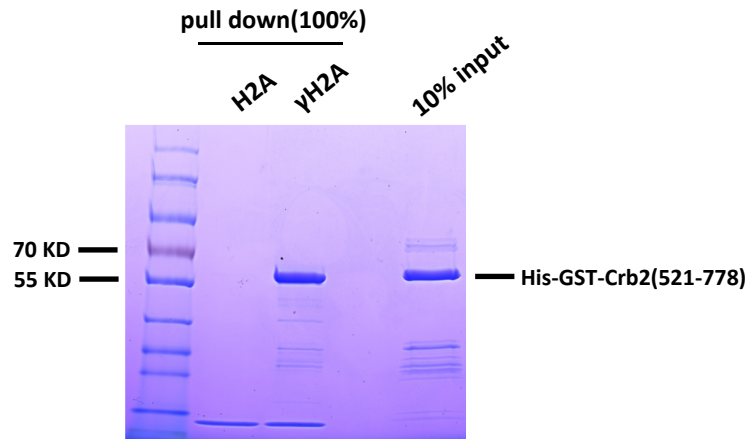

**B**

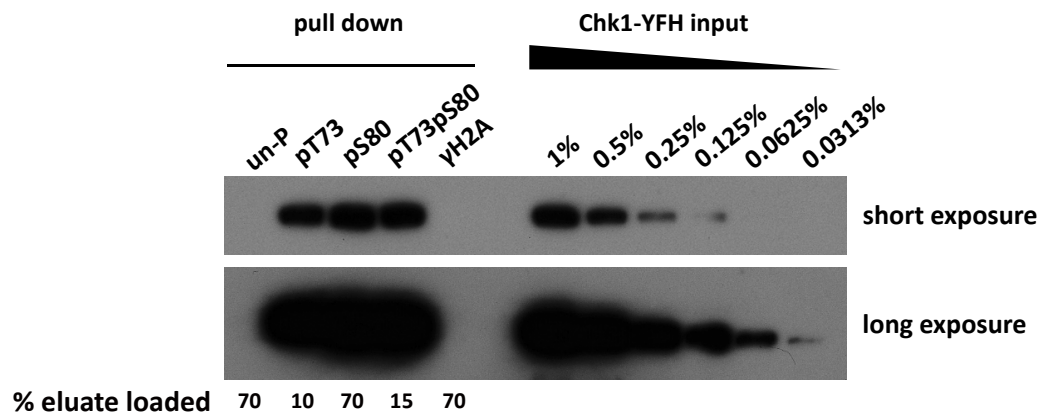

Supplement: Figure S7 — Chk1 is unable to bind the phosphorylated H2A (γH2A) peptide. (A) BRCTs in Crb2 can bind the γH2A peptide. Recombinant His-GST-Crb2(521–778) was purified from E. coli with Ni-NTA beads and incubated with Hta1(120–132) (H2A) or Hta1(120–132-pS129) (γH2A) peptide. Peptides were pulled down by streptavidin Dynabeads and eluted by boiling in SDS loading buffer. The eluates and input were analyzed by 4%–20% SDS-PAGE followed by Coomassie staining. (B) Chk1 cannot bind the γH2A peptide. The experiment was performed as in Figure 4B. (PDF) [file pgen.1002817.s007.pdf]

Qu et al. Figure S8

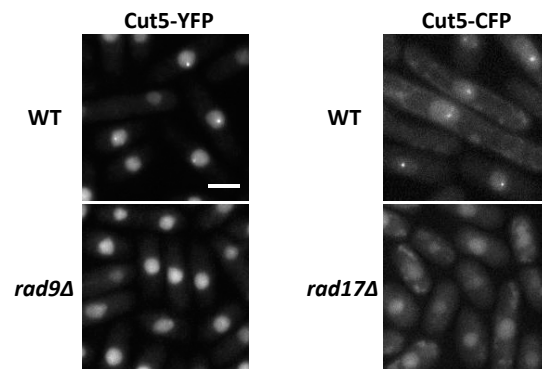

Supplement: Figure S8 — DNA damage-induced Rad4/Cut5 focus formation requires Rad9 and Rad17. Cells expressing Cut5-YFP or Cut5-CFP in WT, rad9Δ or rad17Δ background were treated with 160 Gy IR and then examined by fluorescence microscopy. Strains used were DY6557, DY6558, DY6559 and DY6560. Bar, 5 µm. (PDF) [file pgen.1002817.s008.pdf]

Qu et al. Figure S9

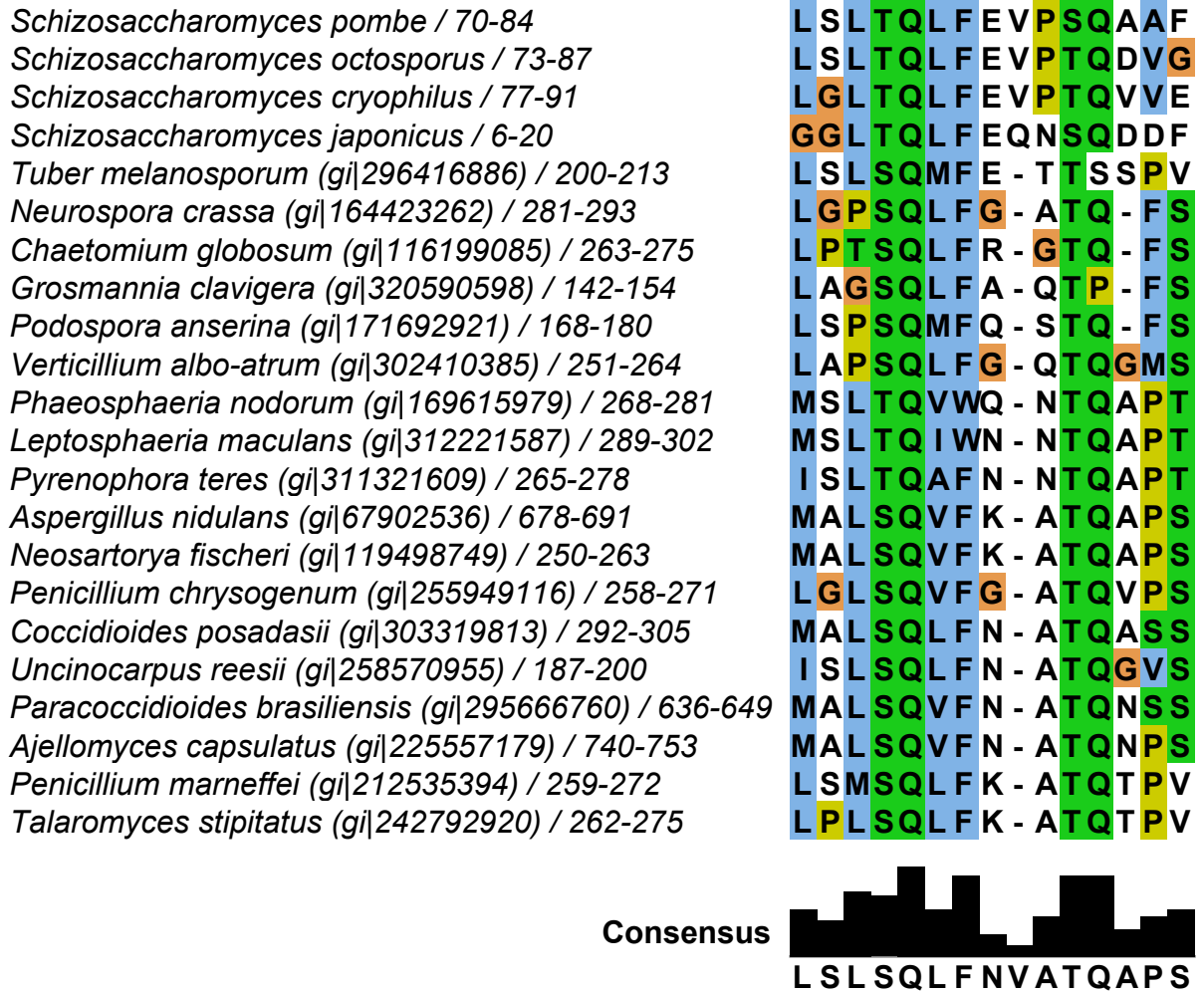

Supplement: Figure S9 — The SQ/TQ cluster region of Crb2 is conserved in its orthologs in Ascomycota fungi species outside of the fission yeast clade. (PDF) [file pgen.1002817.s009.pdf]

Qu et al. Figure S10

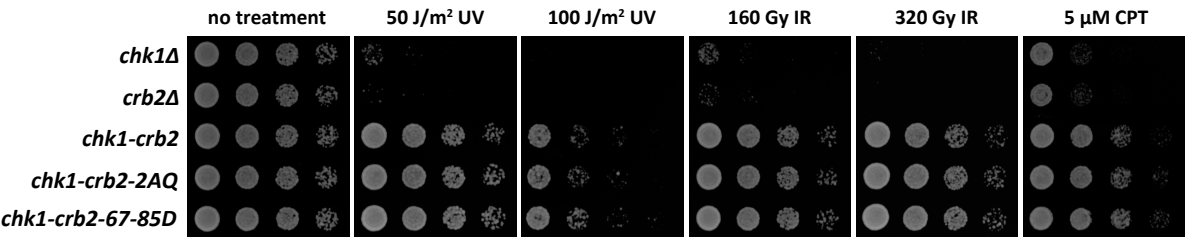

Supplement: Figure S10 — Crb2(67–85) sequence is dispensable when Crb2 is fused with Chk1. Spot assay was performed as in Figure 2B. Strains used were DY809, DY6507, DY6510, DY6511 and DY8046. (PDF) [file pgen.1002817.s010.pdf]
