# Supplementary material for: Phosphorylation-Dependent Interactions between Crb2 and Chk1 Are Essential for DNA Damage Checkpoint
Source: PLoS Genet. 2012 Jul 5;8(7):e1002817. doi: 10.1371/journal.pgen.1002817 (PMC3390401; doi:10.1371/journal.pgen.1002817)
Supplement: Table S1 — Strains used in this study. (DOC) [file pgen.1002817.s011.doc]

**Table S1. Strains used in this study**

| **Strain** | **Mating**  **Type** | **Genotype** |
| --- | --- | --- |
| DY6495 | *h+* | *leu1-32 ura4-D18 chk1-GFP::ura4+ rad22-mCherry::kanMX crb2∆::ura4+(delete EcoRV)::2xCFP-crb2(leu1+) rad9::ura4+* |
| DY6496 | *h+* | *leu1-32 ura4-D18 his3-D1 ade6-M210(?) chk1-GFP::ura4+ rad22-mCherry::kanMX rad3::LEU2* |
| DY6497 | *h-* | *leu1-32 ura4-D18 crb2∆::natMX chk1-GFP::ura4+ rad22-mCherry::kanMX* |
| DY6498 | *h-* | *leu1-32 ura4-D18 chk1-GFP::ura4+ rad22-mCherry::kanMX crb2∆::ura4+(delete EcoRV)::2xCFP-crb2(leu1+)* |
| DY6499 | *h+* | *leu1-32 ura4-D18 his3-D1 crb2(1-358)-LZ chk1-GFP::ura4+ rad22-mCherry::kanMX* |
| DY6500 | *h-* | *ura4-D18 crb2∆::natMX chk1-GFP::ura4+ rad22-mCherry::kanMX leu1-32::crb2(276-778)(leu1+)* |
| DY6502 | *h-* | *ura4-294 his3-D1 lys1-131::dis1-mCherry-LacI(lys1+) erg7ter::lacOrepeat(ura4+) arg3::HOsite-natMX(arg3-) ars1::pJR1-41XH+HO(his3+) rad22-2xCFP::kanMX leu1::chk1pro-chk1-YFH(leu1+)* |
| LD195 | *h-* | *leu1-32 ura4-D18 crb2∆::ura4+ chk1-9myc2HA6His::ura4+* |
| LD346 | *h+* | *leu1-32 ura4-D18 chk1∆::ura4+* |
| DY377 | *h-* | *ura4-D18 crb2∆::ura4+ chk1-9myc2HA6His::ura4+ leu1-32::crb2(leu1+)* |
| DY369 | *h-* | *ura4-D18 crb2∆::ura4+ chk1-9myc2HA6His::ura4+ leu1-32::crb2-T73A(leu1+)* |
| DY370 | *h-* | *ura4-D18 crb2∆::ura4+ chk1-9myc2HA6His::ura4+ leu1-32::crb2-S80A(leu1+)* |
| DY371 | *h-* | *ura4-D18 crb2∆::ura4+ chk1-9myc2HA6His::ura4+ leu1-32::crb2-2AQ(leu1+)* |
| DY6503 | *h-* | *ura4-D18 crb2∆::natMX chk1-GFP::ura4+ rad22-mCherry::kanMX leu1-32::2xCFP-crb2(leu1+)* |
| DY6504 | *h-* | *ura4-D18 crb2∆::natMX chk1-GFP::ura4+ rad22-mCherry::kanMX leu1-32::2xCFP-crb2-T73A(leu1+)* |
| DY6505 | *h-* | *ura4-D18 crb2∆::natMX chk1-GFP::ura4+ rad22-mCherry::kanMX leu1-32::2xCFP-crb2-S80A(leu1+)* |
| DY6506 | *h-* | *ura4-D18 crb2∆::natMX chk1-GFP::ura4+ rad22-mCherry::kanMX leu1-32::2xCFP-crb2-2AQ(leu1+)* |
| DY809 | *h-* | *leu1-32 ura4-D18 chk1∆::kanMX* |
| DY6507 | *h+* | *leu1-32 ura4-D18 crb2∆::natMX* |
| DY6508 | *h+* | *ura4-D18 crb2∆::natMX leu1-32::crb2(leu1+)* |
| DY6509 | *h+* | *ura4-D18 crb2∆::natMX leu1-32::crb2-2AQ(leu1+)* |
| DY6510 | *h+* | *ura4-D18 crb2∆::natMX leu1-32::chk1-crb2(leu1+)* |
| DY6511 | *h+* | *ura4-D18 crb2∆::natMX leu1-32::chk1-crb2-2AQ(leu1+)* |
| DY8046 | *h+* | *ura4-D18 crb2∆::natMX leu1-32::chk1-crb2-67-85D(leu1+)* |
| DY485 | *h-* | *ura4-D18 chk1∆::kanMX leu1-32::nmt1-chk1-YFH(leu1+)* |
| DY6512 | *h+* | *leu1-32 ura4-D18 chk1-GFP::ura4+ cut5-CFP::ura4+ crb2∆::natMX* |
| DY6513 | *h+* | *leu1-32 ura4-D18 chk1-GFP::ura4+ cut5-crb2(67-85)-CFP::ura4+ crb2∆::natMX* |
| DY6514 | *h+* | *leu1-32 ura4-D18 chk1-GFP::ura4+ cut5-crb2(67-85-2AQ)-CFP::ura4+ crb2∆::natMX* |
| DY6546 | *h-* | *leu1-32 ura4-D18 cdc25-22 cut5-CFP::ura4+* |
| DY6547 | *h?* | *leu1-32 ura4-D18 cdc25-22 crb2∆::ura4+ cut5-CFP::ura4+* |
| DY6548 | *h-* | *leu1-32 ura4-D18 cdc25-22 crb2∆::ura4+ cut5-crb2(67-85)-CFP::ura4+* |
| DY6549 | *h+* | *leu1-32 ura4-D18 cdc25-22 crb2∆::ura4+ cut5-crb2(67-85-2AQ)-CFP::ura4+* |
| DY6517 | *h+* | *leu1-32 ura4-D18 chk1-GFP::ura4+* |
| DY6518 | *h+* | *leu1-32 ura4-D18 chk1-GFP::ura4+ cut5-CFP::ura4+* |
| DY6519 | *h+* | *leu1-32 ura4-D18 chk1-GFP::ura4+ cut5-crb2(67-85)-CFP::ura4+* |
| DY6520 | *h+* | *leu1-32 ura4-D18 chk1-GFP::ura4+ cut5-crb2(67-85-2AQ)-CFP::ura4+* |
| DY6521 | *h+* | *leu1-32 ura4-D18 crb2∆::ura4+ chk1-9myc2HA6His::ura4+ cut5-CFP::ura4+* |
| DY6522 | *h-* | *leu1-32 ura4-D18 chk1-9myc2HA6His::ura4+ cut5-CFP::ura4+* |
| DY6523 | *h-* | *leu1-32 ura4-D18 crb2∆::ura4+ chk1-9myc2HA6His::ura4+ cut5-crb2(67-85)-CFP::ura4+* |
| DY6524 | *h-* | *leu1-32 ura4-D18 crb2∆::ura4+ chk1-9myc2HA6His::ura4+ cut5-(67-85-2AQ)-CFP::ura4+* |
| DY6525 | *h-* | *ura4-D18 crb2∆::natMX chk1-mCherry::kanMX6 leu1-32::2xYFP-crb2(67-85+276-778)(leu1+)* |
| DY6526 | *h-* | *ura4-D18 crb2∆::natMX chk1-mCherry::kanMX6 leu1-32::2xYFP-crb2(1-358)-LZ(leu1+)* |
| DY6527 | *h-* | *ura4-D18 crb2∆::natMX chk1-mCherry::kanMX6 leu1-32::2xYFP-crb2(276-778)(leu1+)* |
| DY6528 | *h-* | *ura4-D18 crb2∆::natMX chk1-mCherry::kanMX6 leu1-32::2xYFP-crb2(1-778)(leu1+)* |
| DY6530 | *h-* | *ura4-D18 crb2∆::ura4+ chk1-9myc2HA6His::ura4+ leu1-32::2xYFP-crb2(1-778)(leu1+)* |
| DY6531 | *h-* | *ura4-D18 crb2∆::ura4+ chk1-9myc2HA6His::ura4+ leu1-32::2xYFP-crb2(1-358)-LZ(leu1+)* |
| DY6532 | *h-* | *ura4-D18 crb2∆::ura4+ chk1-9myc2HA6His::ura4+ leu1-32::2xYFP-crb2(276-778)(leu1+)* |
| DY6533 | *h-* | *ura4-D18 crb2∆::ura4+ chk1-9myc2HA6His::ura4+ leu1-32::2xYFP-crb2(67-85+276-778)(leu1+)* |
| DY6529 | *h-* | *leu1-32 ura4-D18 crb2∆::natMX chk1-mCherry::kanMX6* |
| DY6534 | *h-* | *leu1-32 ura4-D18 crb2∆::natMX chk1-GFP::ura4+ rad22-crb2(67-85)-mCherry::kanMX* |
| DY6535 | *h-* | *leu1-32 ura4-D18 crb2∆::natMX chk1-GFP::ura4+ rad22-crb2(67-85-2AQ)-mCherry::kanMX* |
| DY6536 | *h-* | *leu1-32 ura4-D18 crb2∆::natMX chk1-GFP::ura4+ rad22-mCherry::kanMX* |
| DY6989 | *h-* | *leu1-32 ura4-D18 crb2∆::natMX chk1-GFP::ura4+ rad22-crb2(67-85)-mCherry::kanMX rad9::ura4+* |
| DY6538 | *h-* | *leu1-32 ura4-D18 crb2∆::natMX chk1-GFP::ura4+ rad22-crb2(67-85)-mCherry::kanMX rad3::ura4+* |
| DY6550 | *h-* | *leu1-32 ura4-D18 chk1-9myc2HA6His::ura4+ rad22-mCherry::kanMX cdc25-22 crb2∆::ura4+* |
| DY6551 | *h-* | *leu1-32 ura4-D18 chk1-9myc2HA6His::ura4+ rad22-mCherry::kanMX cdc25-22* |
| DY6552 | *h-* | *leu1-32 ura4-D18 chk1-9myc2HA6His::ura4+ rad22-crb2(67-85)-mCherry::kanMX cdc25-22 crb2∆::ura4+* |
| DY6553 | *h-* | *leu1-32 ura4-D18 chk1-9myc2HA6His::ura4+ rad22-crb2(67-85-2AQ)-mCherry::kanMX cdc25-22 crb2∆::ura4+* |
| DY6554 | *h?* | *leu1-32 ura4-D18 chk1-9myc2HA6His::ura4+ rad22-crb2(67-85)-mCherry::kanMX cdc25-22 crb2∆::ura4+ rad9::ura4+* |
| DY6555 | *h?* | *leu1-32 ura4-D18 rad22-(67-85)-mCherry::kanMX cdc25-22 crb2∆::natMX chk1∆::ura4+* |
| DY6539 | *h-* | *leu1-32 ura4-D18 chk1-GFP::ura4+* |
| DY6540 | *h-* | *leu1-32 ura4-D18 chk1-GFP::ura4+ rad22-mCherry::kanMX* |
| DY6541 | *h-* | *leu1-32 ura4-D18 chk1-GFP::ura4+ rad22-crb2(67-85)-mCherry::kanMX* |
| DY6543 | *h-* | *leu1-32 ura4-D18 chk1-GFP::ura4+ rad22-crb2(67-85-2AQ)-mCherry::kanMX* |
| DY6544 | *h?* | *leu1-32 ura4-D18 crb2∆::natMX chk1-GFP::ura4+ rad22-crb2(67-85)-mCherry::kanMX cut5-T401* |
| DY6534 | *h-* | *leu1-32 ura4-D18 crb2∆::natMX chk1-GFP::ura4+ rad22-crb2(67-85)-mCherry::kanMX* |
| DY6501 | *h?* | *leu1-32 ura4-D18 chk1-GFP::ura4+ crb2∆::ura4+(delete EcoRV)::2xCFP-crb2(leu1+) rad22-mCherry::kanMX cut5-T401* |
| DY6557 | *h-* | *leu1-32 ura4-D18 rad17::kanMX chk1-GFP::ura4+ cut5-CFP::ura4+* |
| DY6558 | *h+* | *leu1-32 ura4-D18? rad9::ura4+ cut5-YFH::leu1+* |
| DY6559 | *h-* | *leu1-32 ura4-D18? rad9-CFP::ura4+ cut5-YFH::leu1+* |
| DY6560 | *h+* | *leu1-32 ura4-D18 cut5-CFP::ura4+* |
| DY376 | *h-* | *ura4-D18 crb2∆::ura4+ chk1-9myc2HA*6His::ura4+ *leu1-32::crb2(leu1+)* |
| DY383 | *h-* | *ura4-D18 crb2∆::ura4+ chk1-9myc2HA6His::ura4+ leu1-32::crb2-2AQ(leu1+)* |
| DY6561 | *h-* | *leu1-32 ura4-D18 crb2∆::ura4+ chk1-9myc2HA6His::ura4+ rad22-crb2(67-85)-mCherry-Flag::kanMX* |
| DY6562 | *h-* | *leu1-32 ura4-D18 crb2∆::ura4+ chk1-9myc2HA6His::ura4+ rad22-mCherry-Flag::kanMX* |
| DY6563 | *h-* | *leu1-32 ura4-D18 crb2∆::ura4+ chk1-9myc2HA6His::ura4+ rad22-crb2(67-85-2AQ)-mCherry-Flag::kanMX* |
| DY6565 | *h-* | *leu1-32 ura4-D18 crb2∆::ura4+ chk1-9myc2HA6His::ura4+ rad22-crb2(67-85)-mCherry-Flag::kanMX rad3::ura4+* |
| DY6839 | *h-* | *ura4-D18 crb2∆::ura4+ chk1-9myc2HA6His::ura4+ leu1-32::2xFlag-crb2(1-778)(leu1+)* |
| DY6840 | *h-* | *ura4-D18 crb2∆::ura4+ chk1-9myc2HA6His::ura4+ leu1-32::2xFlag-crb2(1-778-8AQ)(leu1+)* |
| DY6841 | *h-* | *ura4-D18 crb2∆::ura4+ chk1-9myc2HA6His::ura4+ leu1-32::2xFlag-crb2(1-778-8AQ+T73A)(leu1+)* |
| DY6842 | *h-* | *ura4-D18 crb2∆::ura4+ chk1-9myc2HA6His::ura4+ leu1-32::2xFlag-crb2(1-778-8AQ+S80A)(leu1+)* |
| DY6843 | *h-* | *ura4-D18 crb2∆::ura4+ chk1-9myc2HA6His::ura4+ leu1-32::2xFlag-crb2(1-778-8AQ+2AQ)(leu1+)* |
| DY6844 | *h-* | *ura4-D18 crb2∆::ura4+ chk1-9myc2HA6His::ura4+ leu1-32::2xFlag-crb2(1-778-S666R)(leu1+)* |
| DY6845 | *h-* | *ura4-D18 crb2∆::ura4+ chk1-9myc2HA6His::ura4+ leu1-32::2xFlag-crb2(1-778-2AQ)(leu1+)* |
| DY295 | *h?* | *ura4-D18 his3-D1 crb2∆::ura4+ arg3::HOsite-natMX ars1::pJR1-41XH+HO(his3+) rad22-2xCFP::kanMX*  *leu1-32::2xYFP-crb2(1-358)-LZ(leu1+)* |
| DY303 | *h?* | *ura4-D18 his3-D1 crb2∆::ura4+ arg3::HOsite-natMX ars1::pJR1-41XH+HO(his3+) rad22-2xCFP::kanMX*  *leu1-32::2xYFP-crb2(1-358-2AQ)-LZ(leu1+)* |
| LD412 | *h-* | *ura4-D18 his3-D1 crb2∆::ura4+ leu1-32::TAP-crb2(leu1+)* |
| LD2 | *h+* | *leu1-32* |
| DY8362 | *h-* | *ura4-D18 his3-D1crb2∆::ura4+ cdc25-22 leu1-32:: 2xCFP-crb2(leu1+)* |
| DY8363 | *h-* | *ura4-D18 his3-D1crb2∆::ura4+ cdc25-22 leu1-32:: 2xCFP-crb2-T73A(leu1+)* |
| DY8365 | *h-* | *ura4-D18 his3-D1crb2∆::ura4+ cdc25-22 leu1-32:: 2xCFP-crb2-S80A(leu1+)* |
| DY8367 | *h-* | *ura4-D18 his3-D1crb2∆::ura4+ cdc25-22 leu1-32:: 2xCFP-crb2-2AQ(leu1+)* |
| LD715 | *h-* | *ura4-D18 his3-D1crb2∆::ura4+ cdc25-22 leu1-32* |
